# Assessing Global Transcriptome Changes in Response to *South African Cassava Mosaic Virus* [ZA-99] Infection in Susceptible *Arabidopsis thaliana*

**DOI:** 10.1371/journal.pone.0067534

**Published:** 2013-06-27

**Authors:** Erica J. Pierce, M. E. Chrissie Rey

**Affiliations:** School of Molecular and Cell Biology, University of the Witwatersrand, Johannesburg, South Africa; NIAID, United States of America

## Abstract

In susceptible plant hosts, co-evolution has favoured viral strategies to evade host defenses and utilize resources to their own benefit. The degree of manipulation of host gene expression is dependent on host-virus specificity and certain abiotic factors. In order to gain insight into global transcriptome changes for a geminivirus pathosystem, *South African cassava mosaic virus* [ZA:99] and *Arabidopsis thaliana*, 4×44K Agilent microarrays were adopted. After normalization, a log2 fold change filtering of data (p<0.05) identified 1,743 differentially expressed genes in apical leaf tissue. A significant increase in differential gene expression over time correlated with an increase in SACMV accumulation, as virus copies were 5-fold higher at 24 dpi and 6-fold higher at 36 dpi than at 14 dpi. Many altered transcripts were primarily involved in stress and defense responses, phytohormone signalling pathways, cellular transport, cell-cycle regulation, transcription, oxidation-reduction, and other metabolic processes. Only forty-one genes (2.3%) were shown to be continuously expressed across the infection period, indicating that the majority of genes were transient and unique to a particular time point during infection. A significant number of pathogen-responsive genes were suppressed during the late stages of pathogenesis, while during active systemic infection (14 to 24 dpi), there was an increase in up-regulated genes in several GO functional categories. An adaptive response was initiated to divert energy from growth-related processes to defense, leading to disruption of normal biological host processes. Similarities in cell-cycle regulation correlated between SACMV and *Cabbage leaf curl virus* (CaLCuV), but differences were also evident. Differences in gene expression between the two geminiviruses clearly demonstrated that, while some global transcriptome responses are generally common in plant virus infections, temporal host-specific interactions are required for successful geminivirus infection. To our knowledge this is the first geminivirus microarray study identifying global differentially expressed transcripts at 3 time points.

## Introduction

In a compatible host, plant viruses manipulate and recruit host metabolites for translation and replication of their genomes and silence host responses through suppressors, despite attempts by the host to mount a defense response [1], [2], [3], [4], [5], [6], [7], [8], [9]. Virus infection causes host cells to over- or under-express certain pathways, causing both physiological and phenotypic changes in the host [3], [4], [6], [7], [9], [10], [11]. The degree of transcriptome change that a particular host undergoes will change spatially and temporally, and will depend on the compatibility and adaptibility of the pathogen. This host-genotype combination thus determines the severity and type of symptoms displayed [5], [6], [7], [12]. Disease formation is the outcome once a virus has successfully completed genome replication, spread through the plasmodesmata to neighbouring cells and colonised distal tissues by vascular dependent long-distance movement in the host plant [5], [13], [14].

Viral proteins are able to accumulate to much higher levels than host proteins in order to fufill their required tasks in replication, movement and suppression of host defences [4]. This in turn has a huge impact on host cells and causes abnormalities in plant growth and development. Not all changes in host gene expression and metabolism are initiated by specific interactions between virus and host proteins, and alterations can also be consequences of general accumulation of viral proteins and subversion of cellular components [3]. Plant viruses are biotrophic pathogens which cause alterations (either by induction or repression) to a wide array of cellular processes, at transcriptional, translational or posttranslational levels [15]. These processes include, among others, hormonal regulation, cell-cycle control and endogenous transport of macromolecules [3], [4], [6], [7], [9], [10], [16]. From an evolutionary perspective, a constant battle between plant defense and virus infection exists. Plants are capable of counteracting the effects of virus attack with pre-existing physical and chemical barriers (constitutive defense), which if overcome by the virus, activate signalling pathways (induced responses) as the next line of defense. Constitutive (preformed) defences are usually non-specific and are effective against a wide array of abiotic and biotic stresses. Induced responses are more targeted and are triggered upon herbivorous insect or microbial pathogen attack. These specific responses are co-ordinated by defense-related hormones involved in signalling pathways [3], [4], [10], [16], [17]. Upon pathogen attack, induced defences rely on energy resources which are critical to plant fitness. In order to minimise fitness costs and maximise defense responses, plants possess regulatory mechanisms to coordinate pathogen-specific defense responses, which involve signalling molecules that act systemically throughout the plant [18]. Salicyclic acid (SA), jasmonic acid (JA) and ethylene (ET) are the main signalling pathways responsible for regulating responses to biotic and abiotic stresses. In addition, abscissic acid (ABA), auxins, cytokinins, gibberellins, and brassinosteroids have also been implicated [18], [19]. Once activated, these signalling molecules are responsible for reallocating resources away from plant growth and development towards defense. The specificity of plant defense responses is determined by the quantity, composition, and timing of these signal molecules and varies across plant species. The replication and defense strategy of the pathogen determines which defense-related genes are triggered by the plant [18], [19]. Following pathogen infection, antagonistic or synergistic cross talk between signalling pathways enables the plant to devise optimal resistance strategies in order to minimise fitness costs and activate specific defenses. Generally, SA-mediated defenses are usually induced by biotrophic pathogens, whereas necrotrophic pathogens and herbivorous insects are more sensitive to JA/ET mediated defenses [18]. Pathogens on the other hand, are also capable of manipulating these signalling networks as well as suppressing induced defenses for their own benefit, resulting in host susceptibility [16], [18].

*South African cassava mosaic virus* [ZA:99] (SACMV) infects an important food security crop, cassava (*Manihot esculenta* Crantz), in Sub-Saharan Africa, and causes extensive damage to the crop, resulting in Cassava mosaic disease (CMD) [20]. SACMV is a member of the genus *Begomovirus*, and belongs to the *Geminiviridae* family, whose members are transmitted by the whitefly, *Bemisia tabaci* (Gennadius) [21], [22]. Its genome is bipartite, consisting of a DNA-A and DNA-B segment of 2800 nt and 2760 nt, respectively [20]. The bipartite-genome of SACMV encodes at least four proteins on the DNA- A: the viral strand contains the coat protein (CP or AV1) and the pre-CP (AV2). The complementary strand contains three proteins; AC1, AC2 and AC3 from overlapping open reading frames (ORFs). AC1 is required for initiation of DNA replication and is termed the replication-associated protein (Rep), AC2 (TrAP) activates transcription in both the DNA-A and DNA-B of the viral sense genes, and AC3 is the DNA replication enhancer (REn). DNA-B encodes two proteins, namely BC1 and BV1 which are involved in intracellular, intercellular and systemic virus movement. BC1 is found on the complementary strand and mediates cell-to-cell movement of the virus. BV1 is the nuclear shuttle protein (NSP) which controls movement of viral DNA between the nucleus and cytoplasm [21], [23], [24]. Geminiviruses have been implicated in many host-responsive processes such as transcriptional regulation, DNA replication, control of the cell cycle, cell proliferation and differentiation, and macromolecular trafficking in whole plants [10], [24], [25], [26], [27]. In order to complete infection in a host, geminiviruses need to modify certain host-cell pathways. Such changes include:- modulation of plasmodesmata structure and function, host silencing-related defense mechanisms, interactions with proteins such as NAC-domain (*NAM,ATAF1/ATAF2*, and *CUC2)* containing proteins which are involved in growth and development regulation, host gene expression changes, and retinoblastoma-related (RBR) pathway interference [25], [27], [28], [29], [30].

Global analyses of exceptionally large datasets are emerging from transcriptome, protein-protein interaction and regulatory, developmental and metabolic pathway studies in order to construct networks that systematically categorize function and interaction between molecules or organisms at differing levels of complexities [31]. This rapidly increasing area of systems biology, where networks are formed from underlying signalling and regulatory control, as well as cellular function, is referred to as “interactomics” [32]. While deep sequencing and whole-genome tiling assays have recently become more important technologies in plant biology [33], microarrays and qRT-PCR remain accurate and invaluable tools in expression profiling of host-virus interactions. Plant gene-expression networks have been elucidated through microarray technology by identifying global gene expression changes in a host, infected, in most instances, with positive-sense RNA viruses [3], [4], [5], [6], [7]. In a study by Postnikova and Nemchinov [34], a comparative analysis of all published microarray data sets of compatible interactions in *Arabidopsis*, with 11 plant viruses (9 RNA, 1 ds DNA and one ssDNA geminivirus), showed that there was a greater variety of up-regulated genes as compared with repressed genes in the course of viral pathogenesis. Furthermore, each virus-host interaction is unique in terms of altered expression levels, but at the same time, there are some shared genes affected by all viruses. Only one whole genome microarray gene expression study has been conducted on a DNA geminivirus, *Cabbage leaf curl virus* (CaLCuV), at 12 days post infection (dpi) in *Arabidopsis* [10].

The *Arabidopsis* experimental system remains the host of choice due to its adaptable and favourable genetic nature, and is the most thoroughly studied organism providing readily available community resources. This allows for more interdisciplinary and multi-investigative studies to take place [35]. The *Arabidopsis* interactome, in particular, can provide information about conserved genes likely to be involved in the same biological process across species such as humans (*Homo sapiens)*, yeast (*Saccharomyces cerevisiae*), fruit-fly (*Drosophila melanogaster*), and nematode worm (*Caenorhabditis elegans*). In addition, knowledge of signalling pathways and protein complexes has increased existing *Arabidopsis* experimental data by adding previously unknown proteins into existing networks. Based on the predicted *Arabidopsis* interactome, hypothesis-driven data can be added to the current knowledge of signalling and cellular function without the need of a cost-prohibitive, high-throughput experimental approach to validate data [32].

Since annotation of the cassava genome is currently incomplete (www.phytozome.org), and no transcriptome studies have been carried out in cassava (except for a study conducted by Fregene et al 2004 [36], using serial analysis of gene expression (SAGE) of host-plant resistance to Cassava mosaic disease), the model plant system, *Arabidopsis*, was chosen to conduct a susceptibility study with SACMV. A temporal study across 36 days post infection (3 time points) was performed to identify co-regulated defense and stress mechanisms activated by SACMV for establishing infection, and also to identify transient or persistent genes expressed across the course of infection. Global monitoring of gene expression was essential to distinguish if host alterations were SACMV-specific and/or a general biotic stress response. Results from this study, and correlations with other plant viruses, has provided further insight into the little that is known about geminivirus gene expression changes in compatible hosts. This is the first reported geminivirus gene expression microarray study identifying progressive differential transcription during a compatible time course of infection.

## Materials and Methods

This SACMV-[ZA:99] – *Arabidopsis* microarray study is MIAME compliant and has been deposited in the Gene Expression Omnibus (GEO) of NCBI (www.ncbi.nlm.nih.gov/geo/) [37], [38]. The accession number GSE43282 has been assigned to the project and the data is publicly available.

### Agroinfection of Plants and Virus Detection and Copy Number Determination

*Arabidopsis thaliana* (ecotype Columbia-0) seeds were planted in seed trays containing peat pellets (Jiffy Products International), covered with plastic wrap and placed at 4°C for 1 day to eliminate dormancy and ensure uniform germination. These plants were then transferred to growth chambers (Binder Growth Cabinets) operating at 22°C under a 10 h photoperiod, in a humid environment, at a light intensity of 100 µm^−2^ sec^−1^. In order to acclimatize the plants, two-to-three cuttings were made in the plastic covering approximately 2 weeks after planting. This procedure was repeated daily for ten days in order to maintain humidity and avoid air flow around the plants. Once acclimatized, the plastic covering was removed and plants were fertilized and watered as required, until ready for virus inoculations.

Eight-week-old *Arabidopsis* plants were co-inoculated with full-length head-to-tail SACMV DNA-A and DNA-B dimers [20], mobilized into *Agrobacterium tumefaciens* strain AGL1 according to the improved agroinfection protocol of Pierce et al., unpublished. Briefly, five hundred microlitres of *Agrobacterium* cultures (containing SACMV DNA-A and DNA-B) were separately inoculated into 5 ml of LB (containing a final concentration of 100 µg/ml of Carbenicillin and Kanamycin), and incubated at 30°C overnight. Once an OD_600_ of 1.8/2.0 was reached (approximately 18 h), 4 ml of culture was sub-inoculated into 30 ml LB with antibiotics for approximately 24 h. One millimetre of each culture (OD of 1.8/2.0) was spun down and the supernatant removed. Sterile water was then added, mixed and spun for 1 min. The pellet was then resuspended in 200 µl LB and equal volumes of DNA-A and DNA-B were mixed together. Approximately 100 µl (for a 10 cm high plant) was used to wound the stems by needle puncture, and the inoculum was then injected along the stem, concentrating on the apex. Plants were covered for 2 days and re-acclimatised to adapt to chamber conditions. Healthy control plants were mock-inoculated with AGL1 cultures only. Virus inoculations and harvesting of leaves was done at the same time of day in order to maintain consistency between time points and to minimize variations in gene expression patterns due to abiotic factors.

Total nucleic acid (TNA) was extracted from SACMV-infected and mock-inoculated *Arabidopsis* plants according to the CTAB (cetyltrimethylammonium bromide) method of Doyle and Doyle (1987) [39]. Fifty milligram young leaf samples were ground in liquid nitrogen and TNA was extracted by the addition of 0.5 ml pre-heated CTAB extraction buffer (2% CTAB, 20 mM EDTA, 1.4 M NaCl, 100 mM Tris pH 8.0) and ß-mercaptoethanol (to a final concentration of 0.1% v/v). The aqueous layer containing the TNA was extracted using chloroform:isoamyl (24∶1) in a two-step process and the nucleic acids precipitated with an equal volume of isopropanol. The pellet was then washed with 70% ice-cold ethanol, vacuum dried and resuspended in 50 µl 1 X TE buffer (10 mM Tris pH 8.0, 1 mM EDTA) containing 20 µg/ml RNase A.

PCR was carried out using BV1 primers that amplify a 168 bp region on SACMV DNA-B genome component. BV1 primers consisted of the following sequences: BV1 Forward 5′TACGGCATGCCTAGGTTGAAGGAA3′ and BV1 Reverse 5′ATCCACATCCTTGAACGACGACCA3′. Approximately 1 µg of TNA was added to each reaction consisting of 0.1 volume 10 X Taq buffer (NHSO_4_), 10 mM dNTPs, 0.04 volumes of 25 mM MgCl_2_, and 1.25 U Taq DNA Polymerase, Recombinant (Fermentas) of which 10 µM of each primer was added, making up a final reaction volume of 50 µl. Amplification was carried out utilizing the MyCycler™ Thermal Cycler (Bio-Rad) with cycling conditions programmed for 1 cycle at 95°C for 1 min, followed by 30 cycles at 93°C for 30 sec, 60°C for 30 sec, and 72°C for 30 sec, this was followed by a final extension step for 7 min at 72°C.

In order to determine SACMV copy number, absolute quantification was performed. Rolling circle amplification of SACMV DNA-B was carried out using the Illustra™ Templiphi™ 100 Amplification kit (GE Healthcare) according to the manufacturer’s instructions. A standard curve was constructed (in duplicate) using 5 known concentrations of SACMV DNA-B RCA products spiked with 200 ng of healthy *Arabidopsis* TNA. In order to obtain a curve where SACMV DNA-B was present at 100 000, 10 000, 1000, and 10 copies, the following calculations were followed:

1. Calculating mass of a single DNA-B molecule
  m = (n)(1mole/6,023×10^23^ molecules (bp))(660 g/mole)
   = (n)(1.096×10^−21 ^g/bp)
  Where:
  n = DNA size (bp)
  m = mass
  Avogadro’s no. = 6.023×10^23^ molecules/1 mole
  Average MW of a double-stranded DNA molecule = 660 g/mole
2. Calculating the mass of DNA-B required to achieve the copy no. of interest
  Copy no. of interest x mass of single DNA-B molecule = mass of DNA-B required
  Where copy no. = 100 000, 10000, 1000, 100, and 10 virus copies
  Mass of single DNA-B molecule = that obtained from point 1 above.
3. Calculating the concentration of DNA-B required to achieve copy no. of interest
  Mass (g) (step 2)/volume pipetted in each reaction

The cartridge-purified BV1 primer pair (explained in SACMV detection section) was used for absolute quantification real-time PCR. Quantitative PCR was performed using the Maxima® SYBR Green qPCR Master Mix (2×) kit (Fermentas). Three biological replicates and two technical replicates were carried out at each time point. Target samples were prepared in LightCycler capillaries (Roche Applied Science) containing 10 µl of Maxima® SYBR Green qPCR Master Mix (2×) with a final MgCl_2_ of 2.5 mM, 0.5 mM of each primer, and 2 µl template DNA (200 ng) in a final volume of 20 µl. RCA DNA-B standards were prepared as above with the addition of 200 ng of healthy *Arabidopsis* TNA spiked into each reaction in order for the standards to be homologous to the target samples. Cycling conditions consisted of an activation mode of 95°C for 10 min, followed by 32 amplification cycles run at 95°C for 15 sec, 55°C for 30 sec, and 72°C for 30 sec for a single acquisition (fluorescence detection at 520 nm at the end of the elongation phase for each cycle). A melting curve was then performed by heating to 95°C, cooling to 65°C for 30 sec, and slowly heating to 95°C at 0.1°C/sec with continuous measurement of fluorescence at 520 nm, followed by a final cooling step at 40°C for 10 sec. All quantitative PCR data was analysed using the Roche LightCycler Software Version 4.

VirD2 PCR was carried out in order to detect *A. tumefaciens* AGL1Ti plasmid (TiBo542) presence in healthy and infected *Arabidopsis* leaf samples at 14, 24, and 36 dpi. Primers were designed for the virD2 gene (AF242881) from *A. tumefaciens* AGL1Ti plasmid (TiBo542), containing a C58C1 chromosomal background [40]. This primer pair amplified a 360 bp region of the virD2 gene: virD2 Forward, 5′GCAGAGCGACCAATCACATA3′ and virD2 Reverse, 5′ GGCTTCAGCGACATAGGAAG3′. Approximately 1 µg TNA was added to each reaction consisting of 0.1 volume 10 X Taq buffer (NHSO_4_), 10 mM dNTPs, 0.04 volumes of 25 mM MgCl_2_, and 1.25 U Taq DNA Polymerase, Recombinant (Fermentas) of which 10 mM of each primer was added, making up a final reaction volume of 50 µl. Amplification was carried out utilizing the MyCycler™ Thermal Cycler (Bio-Rad) with cycling conditions: 1 cycle at 95°C for 4 min, followed by 30 cycles at 95°C for 30 sec; annealing temperatures at of 57°C for 30 sec; an elongation step set at 72°C for 30 sec; followed by a final extension step for 4 min at 72°C.

A standard curve was constructed (in duplicate) using 6 known concentrations of AGL1 Ti plasmid, TiBo542, which is approximately 250 kb in size in order to obtain 100 000, 10 000, 1000, 100, 10, and 1 copy(ies), respectively. In order for standards to be as homologous to the target samples as possible, 200 ng of *Arabidopsis* healthy TNA was spiked into each standard. Calculations were carried out as previously described in SACMV copy number determination section. For quantitative PCR, 3 biological replicates were pooled for healthy and SACMV-infected TNA samples, respectively, at each time point (14, 24, and 36 dpi), and a technical replicate was performed for each biological replicate. Samples were prepared in LightCycler capillaries (Roche Applied Science) containing 10µl of Maxima® SYBR Green qPCR Master Mix (2×) with a final MgCl_2_ of 2.5 mM, 0.5 mM of each virD2 primer, and 2 µl template DNA (200 ng) in a final volume of 20 µl. Cycling conditions consisted of an activation mode of 95°C for 10 min, followed by 40 amplification cycles run at 95°C for 15 sec, 57°C for 30 sec, and 72°C for 30 sec for a single acquisition (fluorescence detection at 520 nm at the end of the elongation phase for each cycle). A melting curve was then performed by heating to 95°C, cooling to 65°C for 30 sec, and slowly heating to 95°C at 0.1°C/s with continuous measurement of fluorescence at 520 nm, followed by a final cooling step at 40°C for 10 sec.

### Gene Expression Studies

#### Extraction, purification and quantification of RNA

In order to limit variation in profiling entire organs or tissues, only the rosette leaves closest to the meristem tip, representing cells containing active geminivirus replication) were sampled. Three independent biological replicates and 1 technical replicate (total RNA from biological replicate 1) were carried out. For each biological replicate, total RNA was extracted from pooled SACMV-infected or healthy *Arabidopsis* leaves at 14, 24, and 36 dpi using a QIAzol lysis reagent modified method originally described by Chomczynski and Sacchi 1987 [41]. Uppermost tissue from 2–3 pooled leaves from individual *Arabidopsis* plants in each biological replicate was ground in liquid nitrogen with a mortar and pestle and 1 ml of QIAzol (Qiagen) added. Samples were then incubated at 60°C for 5 min followed by centrifugation at 13400 rpm for 10 min at 4°C. The supernatant was then treated with 200 µl of chloroform, vortexed for 15 sec, left at room temperature (RT) for 2–3 min and centrifuged at 13400 rpm at 4°C for 15 min. The aqueous phase was carefully pipetted into a new tube and precipitated by adding isopropanol and 0.8 M Sodium Citrate/1.2M NaCl (Sigma), half volume of aqueous phase of each. The tubes were then mixed by gentle inversion and incubated for 10 min at RT, followed by another centrifugation step at 13,400 rpm at 4°C for 10 min. The RNA pellet was washed with 75% ice-cold ethanol, vortexed gently, and centrifuged at 10600 rpm at 4°C for 10 min. The supernatant was discarded and centrifuged for a further 10600 rpm at 4°C for 2 min. Samples were dried at 37°C for 5–10 min and resuspended in 50 to 100 µl of sterile water (Sabax water for injections, Adcock Ingram),and placed at 55°C for RNA to dissolve. In order to purify the RNA samples, the RNeasy Mini Protocol for RNA cleanup (Qiagen) was performed according to manufacturer’s instructions (RNeasy ® Mini Handbook, Qiagen), and 0.5 ul of Ribolock RNAse inhibitor (Fermentas) was added to each 50 ul sample (14 and 24 dpi) and 1 ul to 100 ul for 36 dpi samples. Concentration and purity (A_260_/A_280_ and A_260_/A_280_ ratios) of the samples after cleanup was assessed on the Thermo Scientific NanoDrop™ 1000 Spectrophotometer. RNA integrity was pre-assessed on a 1% TBE gel (not shown). Stringent RNA quality control was carried out using the Agilent 2100 Bioanalyzer (Eukaryote Total RNA Pico series II chip, version 2.5) (not shown).

To detect contaminating DNA in the RNA samples, RT-PCR was carried out using primers designed to amplify an exon/intron region from the *Arabidopsis* Ubiquitin gene (AT4G05320). Primer sequences were as follows: - UB Forward 5′ATTTCTCAAAATCTTAAAAACTT3′ and UB Reverse 5′TGATAGTTTTCCCAGTCAAC3′. cDNA synthesis was carried out as follows: - Oligo dT primer (0.5 ug/ul) (Invitrogen), 0.5 ul Ribolock RNAse inhibitor (Fermentas) and RNAse free water were added to 1 ug of total RNA (total volume 11.6 µl) and samples heated to 70°C for 10 min and chilled on ice. A 7.8 µl master mix containing 5 X buffer, MgCl2 (2.5 mM), 10 mM dNTPS, and 1 µl ImProm-II™ enzyme (Promega) was added to each reaction and RT was carried out utilizing the MyCycler™ Thermal Cyler (Bio-Rad) consisting of 1 cycle of 25°C for 10 min, 42°C for 60 min, and 70°C for 15 min. PCR using Ubiquitin primers was carried out using 100 ng (5 µl) of *Arabidopsis* TNA (positive control) and 5 µl of RT product, with RNAse free water as a negative control. Reaction mixtures contained 10 X reaction buffer, 10 µM Ubiquitin F and R primer (0.5 µM each final), and 2.5 U Dream Taq. Amplification was carried out utilizing the MyCycler™ Thermal Cycler (Bio-Rad) with cycling conditions of DNA denaturation and *Taq* DNA Polymerase activation for 20 sec at 95°C, and then 35 cycles of denaturation for 30 sec at 95°C, annealing for 30 sec at 55°C and extension for 60 sec at 72°C. The amplification products were examined by electrophoresis on a 1% agarose gel stained with ethidium bromide (EtBr) to a final concentration of 10 µg/µl in a 1 X TAE electrophoresis buffer containing 50 µg of EtBr run at 75V.

#### RNA amplification, labelling, microarray hybridization and scanning

Total RNA (1 µg) was amplified using the Amino Allyl Message Amp™II aRNA Amplification Kit (Ambion) following manufacturer’s instructions. During a RNA:Dye coupling, 4 µg of RNA was vacuum-dried at 45°C and resuspended in 5 µl of 0.2 M NAHCO3 (pH 9.0) at RT for 20 min. Two microlitres of each dye (Cy5 or Cy3) was added, incubating for 2 h at RT. Dye labelled aRNA purification was carried out using the RNAEASY MinElute Kit (Qiagen). Dye incorporation (into aRNA) was measured using a NanoDrop 1000 Spectrophotomer. Microarray hybridization was carried out according to manufacturer’s instructions (Agilent). One hundred pmol of each cyanine dye, linearly amplified cRNA was added to a hybridization mix containing 10×blocking agent and 25×fragmentation buffer were incubated for 30 min at 60°C to fragment the RNA. Fifty five microliters of 2×GE buffer was then added to the solution, spun gently and placed on ice, ready for hybridization. One hundred and ten microliters of solution was added onto three Agilent 4 X 44 slides containing containing 37,683 *A.thaliana* probes (Version 3), and placed in a rotating hybridization chamber (Agilent) set at 65°C for 18 h. Slides were then washed using Agilent’s Gene Expression Wash Buffers 1 and 2. Briefly, hybridization chambers were disassembled in Wash Buffer 1. The microarray slide was then removed and placed into a 50 ml Greiner tube containing Gene Expression Washer Buffer 1 at room temperature for 1 minute. This step was repeated for each slide (3 times). Each slide was then placed into pre-warmed (37°C) Wash Buffer 2 for 1 minute. Slides were then centrifuged briefly in 50 ml Greiner tubes to remove remaining droplets. Scanning was conducted using a GenePix 4000B scanner (Axon Molecular Devices) at 532 nm for Cy3 and 635 nm for Cy5. Spots were scanned using 5 µm resolution. Adjustments to photomultiplier tubes were made to balance intensities between each dye and to increase signal-to-noise ratios. GenePix Pro 6.0 (Axon Molecular Devices) software was used to quantify spot intensities.

#### Relative quantitative reverse-transcription PCR (qRT-PCR)-microarray validation

cDNA was synthesized from 1 µg of total RNA in a volume of 20 µl using the iScript™ cDNA Synthesis Kit (Bio-Rad) according to the manufacturer’s instructions.

Quantitative RT-PCR was carried out using primer sets selected from the primer library for *Arabidopsis* Pathogen inducible genes (Sigma), and two additional genes, PDF1.2c (AT5G44430) and ERF4 (AT3G15210) were synthesized for analysis. Primers for three normalization genes were selected from the library which included: CBP20 Forward 5′TGTTTCGTCCTGTTCTACTC3′ and Reverse 5′ACACGAATAGGCCGGTCATC3′, ACTIN2 Forward 5′GCAAGTCATCACGATTGGTGC3′ and Reverse 5′GCAACGACCTTAATCTTCATGCTG3′ and UBC Forward 5′TCAAATGGACCGCTCTTATC3′ and Reverse 5′CACAGACTGAAGCGTCCAAG3′. A fourth normalization gene namely, EF1-alpha was cartridge purified and synthesized as follows, Forward 5′GGAGATTGAGAAGGAGCCCAAGTTC3′ and Reverse 5′GTGTGTGTAGATCCGCCACCTC3′. Four reference genes were selected in order to determine the expression stability of each gene through Normfinder [42]. The top-ranked gene would be the resulting gene with the lowest expression value. For time points 14 and 24 dpi respectively, 3 biological replicates were carried out for both healthy and SACMV-infected cDNA. In addition, a technical replicate was run for each biological replicate. A master mix was prepared for each gene using the Maxima® SYBR Green qPCR Master Mix (2×) kit (Fermentas), with 2 µl of cDNA in a final reaction volume of 20 µl. Two negative controls were prepared which included: - a no-template control to ensure that no primer dimer formation was detected, and a no-RT control was included to ensure that no detectable genomic DNA was present in the sample. Standard curves were prepared at both 14 dpi and 24 dpi by pooling equal amounts of both healthy and SACMV-infected cDNA for each time point, respectively. Six dilutions were prepared for each curve containing the following concentrations: 150 ng, 30 ng, 6 ng, 1.2 ng, and 0.24 ng. In order to account for PCR inhibition, 100 pg of the 18S gene from *N. tabacum* (AY079155.1) was spiked into every sample in order to detect a 139 bp amplicon. 18S primer pairs appeared as follows: - Forward 5′GGCAAATAGGAGCCAATGAA3′ and Reverse 5′GGGGTGAACCAAAAGCTGTA3′. Relative quantification real-time RT-PCR reactions were performed on the LightCycler 2.0 System (Roche Applied Science) with thermal cycling conditions consisting of an initial activation step of 95°Cfor 10 min, followed by a cycling step repeated 40 times consisting of 95°C for 15 sec, 65°C for 30 sec, and 72°C for 30 sec with a single fluorescence measurement. A slight amendment to cycling parameters for the 18S spike-in gene consisted of an annealing temperature of 57°C and 30 cycles, differing slightly to the above-mentioned parameters for all other genes tested. A melting curve analysis was then carried out at 95°C for 0 sec, 65°C for 30 sec, and 95°C for 0 sec at a heating rate of 0.1°C per second and a continuous fluorescence measurement. Melting curve analysis was carried out to confirm that the PCR amplicons corresponded to a single cDNA fragment of expected size. A final cooling step was then carried out at 40°C for 10 sec. Crossing Points (CP) were then determined with the LightCycler software version 4.0 (Roche Applied Science). Real-time values were calculated using the relative standard curve method (Applied Biosystems Technical Bulletin). Target quantity (infected leaf material) was determined by interpolating from the standard curve and then dividing by the untreated control (healthy leaf material). Both target quantity and untreated control was normalized to an endogenous control which was determined from the appropriate standard curve. Three biological replicates and two technical replicates were conducted for infected samples and two biological replicates with two technical replicates were performed for healthy, untreated controls. Calculations as follows: Normalized infected sample = target/endogenous control; normalized healthy sample = target/endogenous control; and fold difference in target = normalized target (infected sample)/normalized target (healthy sample).

## Results

### Arabidopsis Infectivity Assay

**Figure 1:**
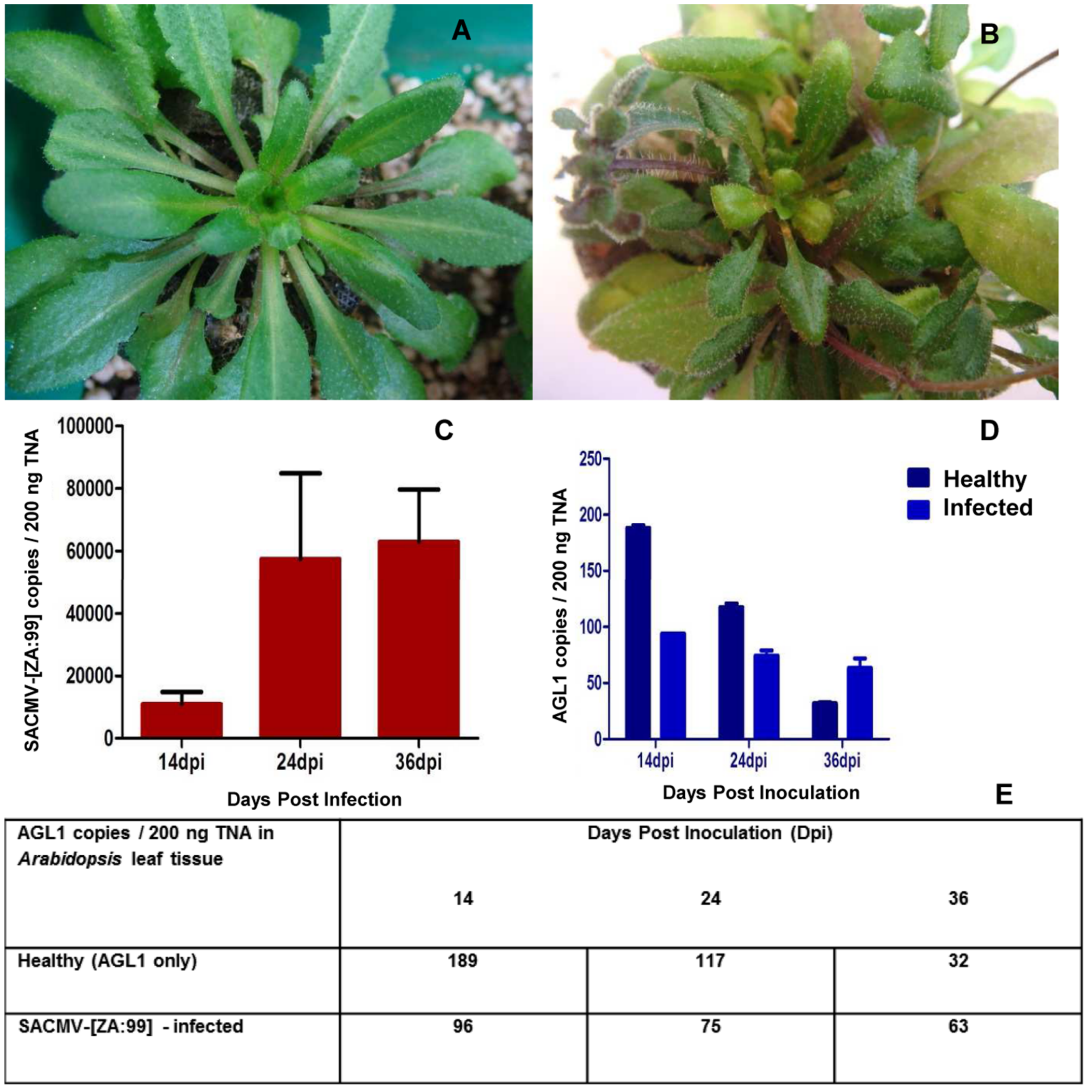
Infectivity assay of SACMV-agroinoculated *Arabidopsis*. **A:** **** Mock-inoculated *Arabidopsis* plants displaying no symptoms (healthy). **B:** SACMV – infected leaves displaying leaf curl and deformation. **C:** SACMV copy number (copies/200 ng TNA) over time. Large error bars indicate variability in virus copy number due to biological differences between replicates. **D and E:** AGL1 detection in 200 ng of TNA from healthy and SACMV – infected leaf tissue across time points 14, 24, and 36 dpi.

Eight-week-old *Arabidopsis* plants were agro-inoculated with SACMV (treatment) and healthy control plants were mock-inoculated with AGL1 cultures to eliminate *Agrobacterium* effects. Symptoms started to appear at 14 dpi and were fully symptomatic at 24 dpi. Overall stunting, slight chlorosis, leaf curl and deformation was observed in infected leaf tissues (Figure 1 B), compared to mock-inoculated controls (Figure 1 A).

Viral DNA accumulation was measured in copy number for 3 biological replicates (independent DNA) and mean values obtained at each time point. BV1 primers were designed for quantitative real-time PCR which amplify a 168 bp region on the SACMV DNA-B component. In 200 ng of total nucleic acid, 1.09×10^4^ SACMV copies were present at 14 dpi, 5.75×10^4^ SACMV copies at 24 dpi, and 6.30×10^4^ SACMV copies at 36 dpi (Figure 1C). Symptom severity thus correlated with an increase in SACMV copy number.

AGL1, although disarmed, is a pathogen capable of causing gene expression changes in a host [43]. In order to confirm host alterations are a consequence of viral infection and not *Agrobacterium* interference, PCR was performed to detect replicating AGL1 in both healthy (inoculated with AGL1 cultures only) and SACMV - infected leaf tissue. AGL1 levels were measured for each biological replicate at 14, 24, and 36 dpi respectively. Although still detected at each time point (Figure 1 D, E), copy number decreased over time, and was negligible at 36 dpi for both mock-inoculated (32 copies remaining) and SACMV- infected (63 copies remaining) plants. AGL1 mock-inoculated controls in the microarray study were used to eliminate the effects of *Agrobacterium* gene expression.

### Microarray Gene Expression Analysis in SACMV-infected *Arabidopsis*

**Figure 2:**
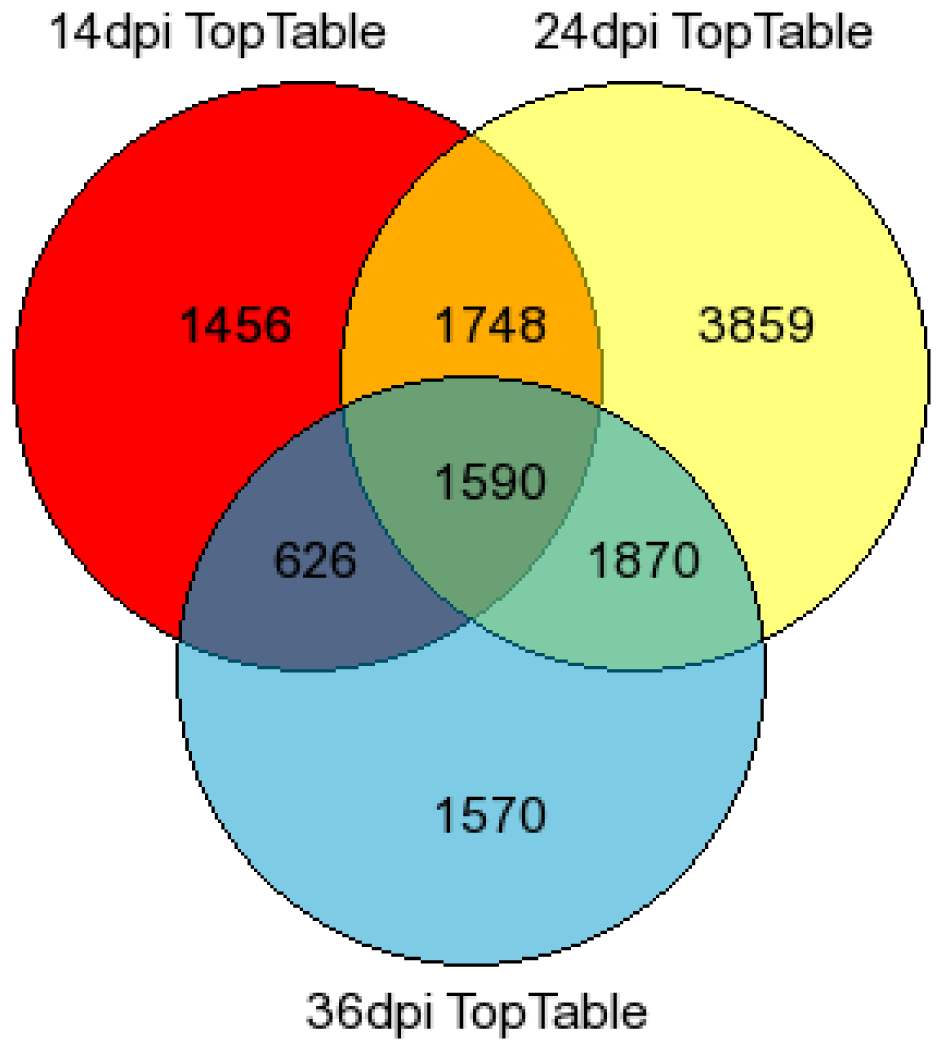
Venn diagram depicting the distribution of 13,934 differentially expressed genes (p<0.05) in SACMV - infected leaf tissue at three time points post infection.

Agilent 4×44k *Arabidopsis* gene expression microarray slides were used to establish global profiles of virus-infected plants at 14, 24, and 36 dpi. Labeled cRNA from three biological replicates and 1 technical replicate were analyzed per time point using a direct comparison experimental design. Fluorescence data obtained from the microarray was imported into Limma (linear models for microarray data) [44] in the R computing environment, where the data was normalized (‘within-array’ global loess normalization and ‘between-array’ quantile normalization), and linear models were fitted in order to contrast SACMV expression values with those of AGL1 mock-inoculated leaf tissue. An output of 13,934 differentially expressed genes was obtained with an adjusted p-value statistic at 0.05 after normalization of data. A total of 1,590 genes were common across the three time points indicated (Figure 2). The number of genes restricted to a particular time point was shown to be 1,456 for 14 dpi, 3 859 for 24 dpi, and 1,570 for 36 dpi indicating unique significant genes at each time point (Figure 2). Gene overlap was highest between 24 and 36 dpi (1,870 corresponding genes), followed by 14 and 24 dpi (1,748 genes showing similarity), with 14 and 36 dpi showing the lowest correlation of 626 genes between the two time points, indicating a large diversion in transcript expression between early and late infection phases. Significantly, maximum levels of gene transcriptional alterations correlated with the peak expression of symptoms, high virus copy number and full systemic virus infection.

Functional categorization of log2-fold induced and repressed genes across 3 time points.

**Figure 3:**
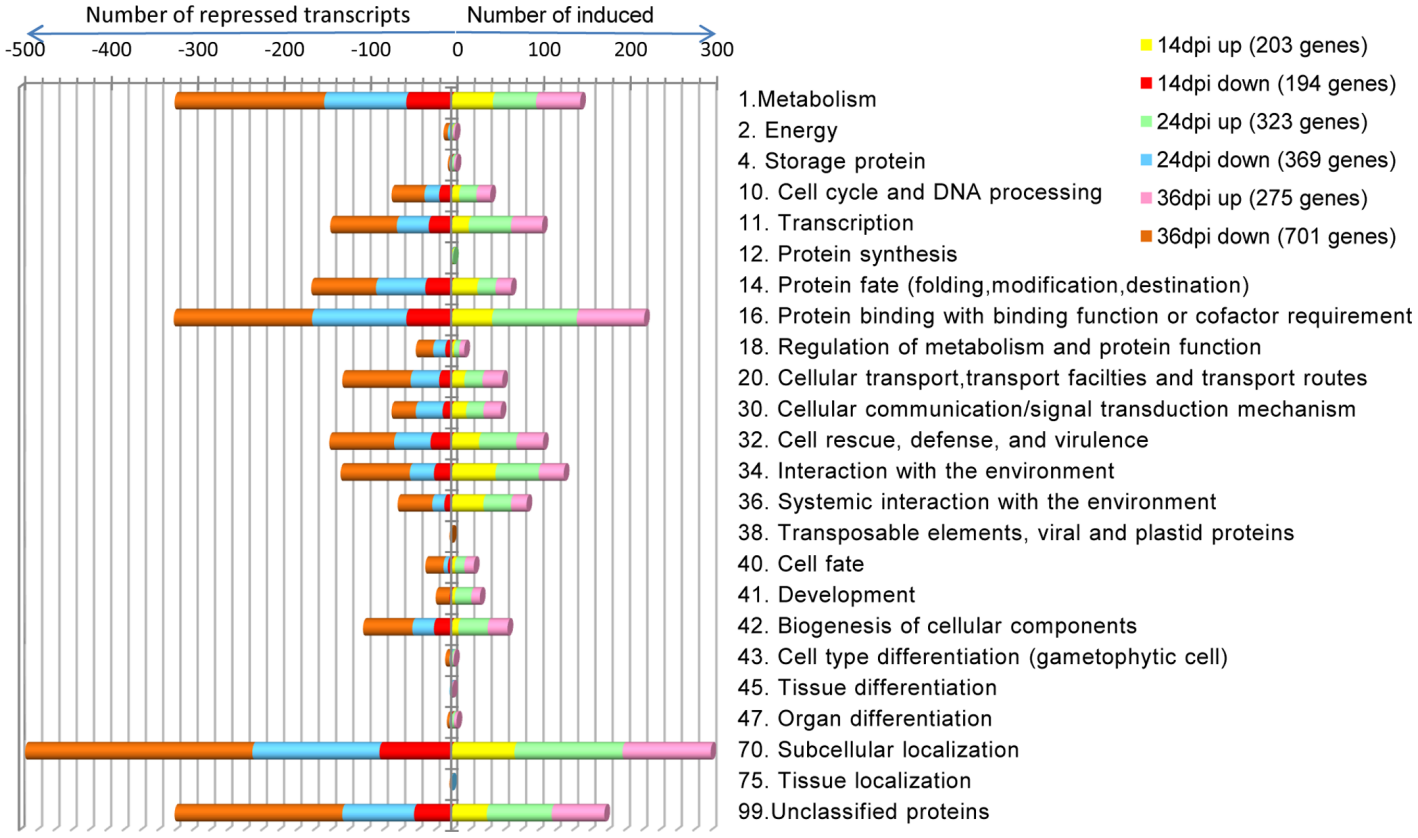
MIPS functional distribution categories of 2-fold differentially expressed transcripts in SACMV - infected ***Arabidopsis*** **leaf tissues at 14, 24 and 36 dpi.**

A log2 fold cut-off (p<0.05) was then applied to the data resulting in a total of 1,743 highly significant differentially expressed genes (Table S1). The fold change expression data was then assigned to a functional category according to the *Arabidopsis* MIPS (Munich Information Centre for Protein Sequence) functional classification scheme (Figure 3). At each time point, MIPS identified the following number of transcripts: - 203 induced and 194 repressed at 14 dpi, 323 induced and 369 repressed at 24 dpi, and 275 induced and 701 repressed for 36 dpi. Based on Fisher’s exact test [45], putative functions for 24 functional categories were established with the majority of differentially regulated transcripts (p<0.05) associated with metabolism, cell cycle and DNA processing, transcription, protein fate (folding, modification, destination), protein binding with binding function or cofactor requirement, cellular transport, transport facilities and transport routes, cellular communication/signal transduction, cell rescue, defense, and virulence, interaction with the environment, systemic interaction with the environment, and sub-cellular localization (Figure 3).

#### Changes in GO functional category expression patterns over the infection period

Examination of the patterns of transcript fold changes in GO functional categories (FCs) (Figure 3) over the infection period revealed some interesting results. For the over- represented FCs such as metabolism (1); transcription (11); protein fate (folding, modification, destination); protein binding (16); cellular transport (20); signal transduction and cell communication (30); defense and cell rescue (32); interaction with the environment; abiotic stress (34 and 36); biogenesis of cellular components (42); and subcellular localization (70) (Figure 3), the trend for each FC was a significant increase (p<0.05) in the total number of differentially regulated (DE) (repressed and induced) genes from onset of symptoms (14 dpi) to 24 dpi and 24 to 36 dpi (establishment of fully systemic symptoms). Of these differentially expressed (DE) transcripts, notably the percentage of repressed genes compared to total number of altered genes in each FC also increased as infection progressed. Several RNA plant virus studies [3], [4] have indicated that in compatible interactions suppression of host transcription defense responses is a pre-requisite for infection, and this study supports previous findings. Additionally, repression of many host-responsive genes at the later stages of pathogenesis when the geminivirus has successfully established systemic infection, may indicate senescence-related responses, and this trend has also been demonstrated in several plant virus-host interactions in *Arabidopsis* [34]. Interestingly, the pattern of change in up-regulated genes in each FC was not as consistent compared with gene down-regulation. A large number of FCs showed that the percentage of induced genes increased from 14 to 24 dpi, and then remained constant or declined in the later stages (36 dpi) of pathogenesis. The GO FCs for cell cycle and DNA processing, transcription, protein binding and biogenesis of cell components, all showed a significant (p<0.05) increase from 14 to 24 dpi, and this is not surprising since all of these functions would need to be induced in order for SACMV to replicate and move systemically during these early to middle stages of acute infection. Defense and cell rescue related transcripts, representing ∼12% of all log2 fold or more differentially expressed genes, while also showing an overall increase in percentage of repressed transcripts across the infection period, interestingly had a steady continuous expression of up-regulated genes (12–16%) over 36 days and did not change significantly. The total number of up-regulated stress/abiotic-related genes (FCs 34 and 36: interaction with the environment; figure 3) declined over the 36 day infection period.

#### Identification of log2 fold induced and repressed genes

**Figure 4:**
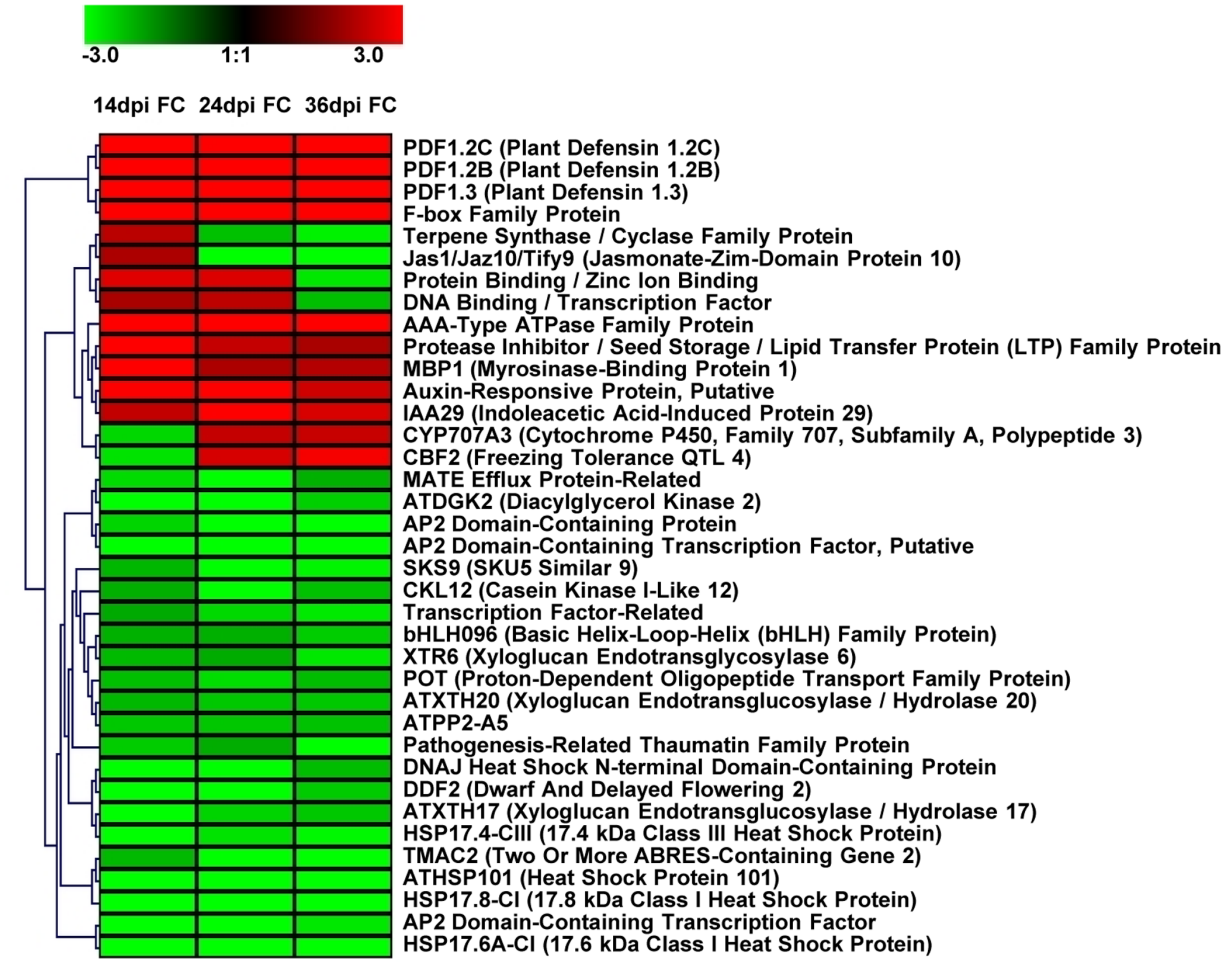
Gene tree heat map showing hierarchical clustering of 37 out of 41 transcripts expressed continuously across time points 14, 24, and 36 dpi (4 unknowns were not displayed). Red bars indicated induction (>2.0) and green bars, repression (<−2.0). Abbreviations: FC (Fold Change).

Once functional categories were established, genes that were continuously expressed across all three time points were identified (Table 1) and a gene tree heat map (Figure 4) was constructed by applying hierarchical clustering using a Euclidean distance metric and average linkage clustering. A total of 41 genes were found to be continuously expressed across time points, 10 showing up-regulation (24.39%), 23 down-regulation (56.10%), 2 down-regulated at 14 dpi then up-regulated at 24 and 36 dpi (4.88%), 4 up-regulated at 14 and 24 dpi, then down-regulated at 36 dpi (9.76%), and 2 up-regulated at 14 dpi then down-regulated at 24 and 36 dpi (4.88%). In addition, we selected the top 20 genes (10 up-regulated and 10 down-regulated) displaying the highest and lowest expression values at each time point to identify which host genes are most reactive to SACMV infection (Table 2). Many transcripts appearing in Table 1 and Figure 4 illustrated that not only were they continuously expressed across time points, but they also appeared in the data listed to have the most highly expressed transcripts (Table 2). Differentially expressed genes were shown to be primarily involved in stress and defense responses as observed with down-regulation of HSP’s (Table S2) and up-regulation of defensins, up-regulation and repression of phytohormone signalling pathways, and induction of genes involved in incompatible reactions, transcription, oxidation-reduction responses and other metabolic processes. An interesting trend observed was the redirection of up-regulated genes, at 14 dpi, that represent many phytohormone signalling responses and related defense responses, towards a large number of induced genes involved in metabolic processes such as oxidation-reduction, transport, and cell-wall modification at 24 and 36 dpi. (Figure 1C,3, and 4, Tables 1 and 2).

**Table 1:** Log2 fold change and adjusted P-values (p<0.05) for 37 transcripts continuously expressed across 3 time points post infection (14, 24, and 36 dpi).

ATG ID	Description	14 dpi Fold Change	14 dpi AdjustedP-value	24 dpi Fold Change	24 dpi AdjustedP-value	36 dpi Fold Change	36 dpi AdjustedP-value
AT5G44430	PDF1.2c (plant defensin 1.2c)	15.82	2.40E-09	10.94	5.80E-202	8.48	4.39E-65
AT2G26020	Arabidopsis thaliana PDF1.2b (plant defensin 1.2b)	14.42	2.40E-09	11.60	2.90E-254	7.91	4.23E-73
AT2G26010	PDF1.3 (plant defensin 1.3)	9.47	2.40E-09	7.25	2.30E-138	6.14	5.48E-47
AT5G07610	F-box family protein	7.48	2.40E-09	7.65	2.40E-175	5.17	1.41E-38
AT2G18193	AAA-type ATPase family protein	4.25	1.14E-08	4.57	9.36E-98	3.51	4.47E-27
AT4G12490	Protease inhibitor/seed storage/lipid transfer protein (LTP) family protein	3.89	2.48E-08	2.32	1.60E-30	2.00	9.44E-09
AT1G52040	MBP1 (MYROSINASE-BINDING PROTEIN 1)	3.07	3.46E-07	2.04	4.82E-22	2.14	2.45E-10
AT4G38860	Auxin-responsive protein, putative	2.98	1.43E-07	3.06	5.39E-53	2.45	4.40E-14
AT4G19700	Protein binding/zinc ion binding	2.66	4.39E-07	2.61	2.89E-39	−2.73	1.62E-17
AT4G32280	IAA29 (indoleacetic acid-induced protein 29)	2.32	1.47E-06	3.27	1.68E-59	2.57	1.60E-15
AT1G61120	Terpene synthase/cyclase family protein	2.22	2.76E-06	−2.28	3.52E-29	−2.86	3.83E-19
AT5G13220	JAS1/JAZ10/TIFY9 (JASMONATE-ZIM-DOMAIN PROTEIN 10)	2.04	2.39E-06	−3.02	7.18E-52	−4.98	6.83E-37
AT5G07580	DNA binding/transcription factor	2.00	1.30E-05	2.24	7.83E-28	−2.26	9.12E-12
AT1G09950	Transcription factor-related	−2.00	3.52E-06	−2.56	1.08E-37	−2.76	6.23E-18
AT5G57015	CKL12 (Casein Kinase I-like 12)	−2.06	2.54E-06	−3.06	5.20E-53	−2.27	5.85E-12
AT1G72210	Basic helix-loop-helix (bHLH) family protein (bHLH096)	−2.09	2.09E-06	−2.08	3.32E-23	−2.42	1.09E-13
AT4G38420	SKS9 (SKU5 Similar 9)	−2.16	1.35E-06	−3.62	5.43E-70	−2.90	1.31E-19
AT5G48070	ATXTH20 (XYLOGLUCAN ENDOTRANSGLUCOSYLASE/HYDROLASE 20)	−2.17	1.63E-06	−2.36	1.48E-31	−2.35	6.23E-13
AT3G02140	TMAC2 (TWO OR MORE ABRES-CONTAINING GENE 2)	−2.19	1.36E-06	−3.90	1.75E-78	−5.99	9.49E-55
AT4G25810	XTR6 (XYLOGLUCAN ENDOTRANSGLYCOSYLASE 6)	−2.20	1.48E-06	−2.03	1.29E-21	−2.73	1.42E-17
AT1G22570	Proton-dependent oligopeptide transport (POT) family protein	−2.26	9.53E-07	−2.62	1.85E-39	−2.22	2.38E-11
AT1G65390	ATPP2-A5 (ATPP2-A5)	−2.36	6.08E-07	−2.35	2.87E-31	−2.29	3.87E-12
AT4G36000	Pathogenesis-related thaumatin family protein	−2.41	7.73E-07	−2.04	5.37E-22	−3.28	2.37E-24
AT5G52020	AP2 domain-containing protein	−2.51	1.32E-06	−5.17	4.00E-114	−3.50	4.96E-27
AT5G45340	CYP707A3 (cytochrome P450, family 707, subfamily A, polypeptide 3)	−2.54	5.57E-07	2.33	1.02E-30	2.36	5.46E-13
AT5G52050	MATE efflux protein-related	−2.60	3.86E-07	−5.16	8.80E-114	−2.08	3.50E-08
AT4G25470	CBF2 (FREEZING TOLERANCE QTL 4)	−2.66	2.03E-07	2.54	3.72E-37	2.92	6.92E-20
AT5G63770	ATDGK2 (DIACYLGLYCEROL KINASE 2)	−3.02	3.06E-07	−4.70	3.80E-101	−2.44	6.02E-14
AT1G74310	ATHSP101 (HEAT SHOCK PROTEIN 101)	−3.09	6.34E-08	−5.18	1.90E-114	−5.47	2.14E-49
AT1G65310	ATXTH17 (XYLOGLUCAN ENDOTRANSGLUCOSYLASE/HYDROLASE 17)	−3.15	6.22E-08	−2.49	1.05E-35	−2.36	4.80E-13
AT2G21510	DNAJ heat shock N-terminal domain-containing protein	−3.30	4.39E-08	−3.58	8.87E-69	−2.23	2.02E-11
AT2G20350	AP2 domain-containing transcription factor, putative	−3.58	3.49E-08	−5.10	4.00E-112	−3.20	2.13E-23
AT1G63030	DDF2 (DWARF AND DELAYED FLOWERING 2) (DDF2)	−3.88	1.63E-08	−3.85	8.35E-77	−2.38	2.78E-13
AT1G54050	17.4 kDa class III heat shock protein (HSP17.4-CIII)	−4.11	1.24E-08	−2.66	8.12E-41	−2.93	5.60E-20
AT1G07400	17.8 kDa class I heat shock protein (HSP17.8-CI)	−5.57	3.11E-09	−4.17	2.22E-86	−5.37	2.09E-40
AT1G22810	AP2 domain-containing transcription factor, putative	−5.96	3.83E-09	−8.59	8.50E-196	−2.74	1.12E-17
AT1G59860	17.6 kDa class I heat shock protein (HSP17.6A-CI)	−6.41	2.40E-09	−6.77	9.00E-155	−3.71	1.13E-24

**Table 2:** Log2 fold change and adjusted P-values (p<0.05) representing the most significantly induced and repressed (10 up- and 10 down-regulated) *Arabidopsis* genes at 14, 24 and 36 dpi.

*Arabidopsis* acc no.	Description	Fold Change	Adjusted P-Value
**14 dpi**			
AT5G44430	PDF1.2c (plant defensin 1.2c) (PDF1.2c)	15.82	2.40E-09
AT2G26020	PDF1.2b (plant defensin 1.2b)	14.42	2.40E-09
AT5G44420	PDF1.2 (Low-molecular-weight cysteine-rich 77)	13.59	2.88E-09
AT2G26010	PDF1.3 (plant defensin 1.3)	9.47	2.40E-09
AT5G07610	F-box family protein	7.48	2.40E-09
AT5G24780	VSP1 (VEGETATIVE STORAGE PROTEIN 1); acid phosphatase (VSP1)	4.78	2.05E-08
AT4G38840	Auxin-responsive protein, putative	4.68	5.26E-09
AT4G25110	ATMC2 (METACASPASE 2)	4.60	6.34E-09
AT1G52400	BGL1 (BETA-GLUCOSIDASE HOMOLOG 1); hydrolase, hydrolyzing O-glycosyl compounds (BGL1)	4.57	4.13E-08
AT2G39030	GCN5-related N-acetyltransferase (GNAT) family protein	4.37	3.49E-08
AT5G13700	APAO/ATPAO1 (POLYAMINE OXIDASE 1); FAD binding/polyamine oxidase (APAO/ATPAO1)	−4.30	1.69E-08
AT4G30280	ATXTH18/XTH18 (XYLOGLUCAN ENDOTRANSGLUCOSYLASE/HYDROLASE 18)	−4.53	1.24E-08
AT3G15210	ATERF-4,Ethylene responsive binding factor 4 DNA binding/protein binding/transcription factor/transcriptional repressor	−4.64	1.31E-08
AT2G29370	Tropinone reductase, putative/tropine dehydrogenase, putative	−5.36	3.83E-09
AT2G20630	Protein phosphatase 2C, putative/PP2C, putative	−5.37	3.23E-09
AT1G07400	17.8 kDa class I heat shock protein (HSP17.8-CI)	−5.57	3.11E-09
AT3G27540	Glycosyl transferase family 17 protein	−5.65	2.88E-09
AT1G22810	AP2 domain-containing transcription factor, putative	−5.96	3.83E-09
AT1G59860	17.6 kDa class I heat shock protein (HSP17.6A-CI)	−6.41	2.40E-09
AT5G10100	Trehalose-6-phosphate phosphatase, putative	−7.86	2.40E-09
**24 dpi**			
AT5G45890	SAG12 (SENESCENCE-ASSOCIATED GENE 12); cysteine-type peptidase (SAG12)	13.16	5.01E-281
AT2G26020	PDF1.2b (plant defensin 1.2b)	11.60	2.86E-254
AT5G44430	PDF1.2c (plant defensin 1.2c)	10.94	5.81E-202
AT3G49340	Cysteine proteinase, putative (AT3G49340)	9.42	4.21E-213
AT5G07610	F-box family protein (AT5G07610)	7.65	2.41E-175
AT2G26010	PDF1.3 (plant defensin 1.3) (PDF1.3)	7.25	2.33E-138
AT4G37990	ELI3-2 (ELICITOR-ACTIVATED GENE 3)	5.56	2.31E-124
AT3G44550	Oxidoreductase, acting on the CH-CH group of donors	5.25	3.12E-116
AT2G18193	AAA-type ATPase family protein	4.57	9.36E-98
AT5G44050	ATGEX1/GEX1 (GAMETE EXPRESSED PROTEIN1)	4.50	9.21E-96
AT2G20350	AP2 domain-containing transcription factor, putative	−5.10	4.03E-112
AT5G52050	MATE efflux protein-related (AT5G52050)	−5.16	8.78E-114
AT5G52020	AP2 domain-containing protein	−5.17	3.97E-114
AT1G74310	ATHSP101 (HEAT SHOCK PROTEIN 101); ATP binding/ATPase	−5.18	1.93E-114
AT2G17660	Nitrate-responsive NOI protein, putative (AT2G17660)	−5.25	3.12E-116
AT2G26150	ATHSFA2 (Arabidopsis thaliana heat shock transcription factor A2)	−5.33	2.13E-118
AT1G59860	17.6 kDa class I heat shock protein (HSP17.6A-CI)	−6.77	9.03E-155
AT5G37940	NADP-dependent oxidoreductase, putative	−6.83	2.78E-156
AT1G22810	AP2 domain-containing transcription factor, putative	−8.59	8.46E-196
AT5G37970	S-adenosyl-L-methionine:carboxyl methyltransferase family protein	−10.16	1.36E-227
**36 dpi**			
AT5G44430	PDF1.2c (plant defensin 1.2c)	8.48	4.39E-65
AT2G26020	PDF1.2b (plant defensin 1.2b)	7.91	4.23E-73
AT2G26010	PDF1.3 (plant defensin 1.3)	6.14	5.48E-47
AT1G31690	Copper ion binding	5.38	1.84E-48
AT1G72920	Disease resistance protein (TIR-NBS class), putative	5.25	4.54E-47
AT5G07610	F-box family protein	5.17	1.41E-38
AT5G21960	AP2 domain-containing transcription factor, putative	4.90	2.84E-43
AT2G40610	ATEXPA8 (ARABIDOPSIS THALIANA EXPANSIN A8)	4.55	2.00E-39
AT2G43590	Chitinase, putative	4.00	5.26E-33
AT2G41180	SigA-binding protein-related	3.74	7.49E-30
AT5G22490	Condensation domain-containing protein	−7.85	1.39E-72
AT1G61820	BGLU46; hydrolase, hydrolyzing O-glycosyl compounds	−8.13	5.34E-75
AT2G38240	Oxidoreductase, 2OG-Fe(II) oxygenase family protein	−8.19	5.36E-63
AT1G43160	RAP2.6 (related to AP2 6); DNA binding/transcription factor	−8.21	1.21E-75
AT3G27170	CLC-B (chloride channel protein B); anion channel/voltage-gated chloride channel	−8.28	3.15E-76
AT4G12400	Stress-inducible protein, putative	−8.56	1.25E-78
AT5G01380	Transcription factor	−8.68	1.41E-79
AT3G02550	LBD41 (LOB DOMAIN-CONTAINING PROTEIN 41)	−9.73	3.34E-88
AT5G63450	CYP94B1 (cytochrome P450, family 94, subfamily B, polypeptide 1); oxygen binding	−12.27	2.40E-107
AT3G56700	Male sterility protein, putative	−14.30	9.11E-121

#### Quantitative reverse-transcription PCR (qRT-PCR) (microarray validation)

**Figure 5:**
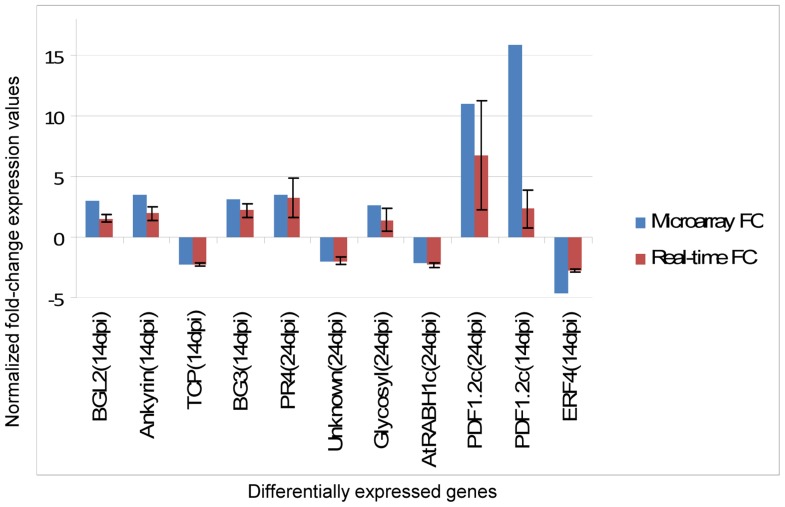
Validation of microarray expression data by relative quantitative real-time RT-PCR (qRT-PCR). Expression changes of 10 selected transcripts depicting similarities in expression patterns between the two technologies are shown. Signal intensities for each transcript were normalized with CBP20 for 14 dpi and Actin2 for 24 dpi. The x-axis represents validated genes at time points 14 and 24 dpi. The y-axis represents normalized fold-change expression values for each transcript. The error bars show standard deviation from 3 biological replicates.

Since the greatest differences in fold-change occurred between 14 and 24 dpi, and 24 dpi was our most significant time point in terms of altered gene expression, we chose to validate expression values obtained from microarray data with relative quantification real-time PCR at these time points (Figure 5). At 14 dpi, 3 up-regulated genes, namely BGL2 (AT3G57260), Ankyrin repeat family protein (AT4G03450), and BG3 (AT3G57240), and two down-regulated genes, Transcription factor family (TCP) (AT2G45680) and Ethylene response factor 4 DNA binding/transcriptional repressor (ERF4)(AT3G15210) confirmed expression results obtained from microarray data. Induced genes such as PR4 (AT3G04720) and Glycosyl hydrolase family 17 protein (AT4G16260) and repressed genes such as an Unknown protein (AT2G32200) and AtRABH1c (AT4G39890) showed similarities to microarray data at 24 dpi. In addition, the plant defensin (PDF1.2c) gene was tested at both 14 and 24 dpi, showing similarities in up-regulation to the microarray data. While fold-change patterns correlated, discrepancies in magnitude between the two platforms is not uncommon, and could be attributed to the differences in normalization methods used, where the use of endogenous controls such as CBP20 at 14 dpi and Actin2 at 24 dpi was carried out for normalization of qRT-PCR data, whereas a global normalization was applied to the microarray data. In addition, cDNA was used for qRT-PCR whereas cRNA was used for microarray analysis, suggesting a more efficient fold-change detection method to changes in gene expression for microarray experiments. All qRT-PCR analyses involved 3 biological replicates for SACMV - infected cDNA and 2 biological replicates for AGL1 mock-inoculated controls.

## Discussion

### Symptom Development and Virus Accumulation in SACMV-infected *Arabidopsis*

*Arabidopsis* plants were observed to be fully symptomatic at 24 dpi, although symptoms started appearing at 12–14 dpi. Symptoms such as stunting of the entire plant, leaf reduction and deformation were observed in all SACMV - infected *Arabidopsis*, while additionally, chlorosis was observed in approximately 60% of infected plants (Figure 1B). SACMV was detected in all infected plants tested. Chlorotic symptoms may be the direct result of the plants attempt to rescue resources from infected tissues via basal resistance mechanisms. If chlorosis is absent in infected tissues, this usually indicates a loss of basal resistance [46], and the appearance of mild chlorosis in the majority (60%) of SACMV – infected Arabidopsis leaves suggests a down-regulation of innate basal resistance leading to expected susceptibility to the virus. An increase in SACMV replication was observed between time points 14 and 24 dpi showing a 5-fold increase. Between 14 and 36 dpi, a 6-fold increase was observed (Figure 1C), confirming that an increase in viral titre correlated with symptom development. These findings were also observed in studies conducted by Babu et al. 2008b [7] in soybean [*Glycine max* (L.) Merr] plants infected with *Soybean mosaic virus* (SMV) whereby at 14 dpi virus titer was approximately 2-fold higher than 7 dpi as detected by Northern hybridizations. Similarly, in a gene expression study conducted by Golem and Culver 2003 [47], a greater fold-change increase was also observed in *Tobacco mosaic virus* (TMV) response genes in *Arabidopsis* Shahdara from 4 dpi to 14 dpi, suggesting that higher levels of TMV were present at a later infection time point.

Previous studies have suggested that *Agrobacterium*, although containing a disarmed plasmid, is able to cause changes in host gene expression but at very early stages of infection. These occur between 3–6 h and 30–36 h after initiation of infection [43]. In order to eliminate the effects of *Agrobacterium* in microarray experiments, *Agrobacterium* mock-inoculated controls are commonly used. In this study, qPCR was conducted on AGL1 mock-inoculated control and SACMV-infected plants to rule out the possibility that *Agrobacterium* was persistently replicating in *Arabidopsis* leaf tissues, consequently causing changes in gene expression. qPCR results showed minimal detectable AGL1 copies, showing a decline from 189 copies (14 dpi) to 32 copies (36 dpi) in mock-inoculated leaf tissue and 96 copies (14 dpi) to 63 (36 dpi) in SACMV- infected leaf tissue (Figure 1D,E). Although *Agrobacterium* AGL1 was still detected by PCR, copy numbers were too low to be considered significant, and most likely represent initial replication following the agroinoculation procedure. Additionally, host gene expression changes in *Arabidopsis* are identified by normalization against mock-inoculated controls, ensuring that alterations are solely due to SACMV.

### Differentially Expressed Transcript Data

Gene expression non-filtered data revealed 13,934 significant (p<0.05) differentially expressed genes (including up- and down-regulated transcripts) in response to SACMV infection at three different time points (14, 24, and 36 dpi). Individual gene transcripts were identified at a particular time point and overlap of genes between time points was also observed (Figure 2). Genes expressed transiently at a particular time point may indicate either induction or repression for a specific function or to conserve energy resources in the host [18], [19], [48], [49]. Those transcripts that appear to show persistent expression (across two or more time points) may be necessary to carry out appropriate function such as stress and defense-like responses for basal resistance to counteract virus attack or alternatively may be induced or repressed by SACMV to aid in its own replication, cell-to-cell spread and systemic movement, as implicated in other studies [4], [10].

As a first step toward assigning differentially expressed genes to function, the distribution of *Arabidopsis* genes significantly induced or repressed at a log2 fold cut-off in SACMV infected *Arabidopsis* leaves were assigned according to the MIPS (http://mips.gsf.de/proj/thal/db/Arabidopsis) classification scheme. For the purpose of this study we refer to early response genes as 14 dpi (initiation of symptoms), to 24 dpi as fully symptomatic, middle-phase genes, and to 36 dpi as late response genes. A general overview of 1,743 differentially expressed transcripts revealed more up-regulated genes (203) than down-regulated genes (194) at 14 dpi, and a higher number of repressed genes for both 24 dpi (369) and 36 dpi (701) compared with induced genes at 24 dpi (323) and 36 dpi (275), respectively. The margin between induced and repressed genes at 14 dpi was very narrow (difference of 9 genes favouring up-regulation) which increased to a 46 gene difference at 24 dpi, favouring down-regulation. At 36 dpi, a 426 difference in down-regulated genes was evident (Figure 3). We propose that the higher number of induced genes at 14 dpi may reflect more of a general non-specific innate host response to virus invasion by the activation of stress and defense-like genes, whereas the increase in down-regulated genes at 24 and 36 dpi is indicative of SACMV attempt to hijack many host processes for its own benefit, leading to repression of a large number of genes. The host (*Arabidopsis*) may also be attempting to divert metabolites such as those involved in, among others, glycolysis and gluconeogenesis, pentose-phosphate pathways, and carbohydrate metabolism, away from normal cell function in order to conserve energy, as well as defend itself from SACMV attack (Figure 3).

### Comparison of 2-fold Gene Expression Patterns with Other Datasets

In a comparative plant virus microarray study by Postinova and Nemchinov [34], they demonstrated that collectively from eleven Arabidopsis-virus interaction studies, 7639 unique genes were significantly changed at least log2 fold, which represents 23% of the *Arabidopsis* genome. SACMV shared 817 genes (across three time points) in common with the 7639 unigenes (Table S3), and 524 genes (across three time points) in common with the geminivirus, CaLCuV, at 12 dpi (Table S4). Only 19 genes (Table S5) were common to SACMV, CaLCuV and the 7639 unigenes [34]. This was not surprising as only 198 genes were differentially expressed in response to all eleven viruses (9 RNA; 1 dsDNA; 1 ssDNA) in the *Arabidopsis* comparative microarray study [34], pointing to the unique nature of virus-host interactions [34]. However, as useful as these comparisons are, one must acknowledge the limitations in comparing individual and combined datasets. Another notable observation was that an estimated 12%, 15% and 22% of responsive genes described in the SACMV, eleven *Arabidopsis*-virus and CaLCuV studies, respectively, were related to abiotic/biotic stress/defense, and over-representation in this functional category is not uncommon in virus-host interactions [3], [4], [34].

In the CaLCuV study [10], at 12 dpi (representing prominent symptoms and active viral replication), a significantly (q value <0.005) high number (5365 representing 23% of the Affymetrix total 22,748 gene probes) of genes were found to be differentially expressed, with 3004 being up-regulated and 2631 down-regulated (6% difference). Similarly in this study, at 14 and 21 dpi, differences in numbers of up-regulated and suppressed genes were not significant, but at 36 dpi there was a significant number of repressed compared with up-regulated genes (difference of 43%). If one compares SACMV at 24 dpi with CaLCuV at 12 dpi (approximate similar stages of infection; fully symptomatic), the number of differentially expressed genes from the total number represented on the arrays, is significantly lower (4% of the Agilent 37,683 array probes) compared with CaLCuV (23%). However, thirty three percent of the 1,743 log2 fold altered transcripts were differentially expressed at 24 dpi in this study, compared with 23% at 12 dpi in CaLCuV-infected *Arabidopsis*. This striking difference in gene expression levels, in the identical host, between two different geminiviruses, is hypothesized to be partly attributed to the more virulent nature of CaLCuV in *Arabidopsis*, resulting in a more severe symptom phenotype, and symptoms appearing much earlier, compared with SACMV. This would point to a greater susceptible host response and a higher number of gene alterations associated with cellular processes redirected by CaLCuV, suggesting that CaLCuV may be less adapted to this non-natural host compared to SACMV. Additionally, we consider it reasonable to speculate that different geographical evolutionary patterns of CaLCuV, a New World northern hemisphere geminivirus, and SACMV (southern hemisphere) from the Old World, in relation to the Arabidopsis, may also contribute to differences in host response.

Forty-one genes (2.3%) at a log2 fold cut-off were present across all three time points in SACMV infected *Arabidopsis* (Table 1), indicating that most genes were transiently expressed and not sustained throughout virus progression in time. A snapshot of the most significant highly induced and repressed (highest expression values) early-response genes occurring at 14 dpi indicated more signalling-related defense responses, whereas those appearing from middle to late responses (24 and 36 dpi) were primarily involved in metabolic functions (Table 2). As the shift continues from early to middle and late gene expression, host metabolism is altered, which suggests that more host metabolites may be diverted to aid in SACMV replication and cell-to-cell-spread, and at the same time, the host is diverting resources away from normal cell functions to minimize fitness costs in an attempt to defend itself against SACMV. At the 24 and 36 post-infection stage, a more specific defense response appears to be induced, evidenced by the induction of putative stress (AT4G12400) and disease resistance (AT1G72920) proteins (Table 2). Results from Table 1 and 2 provide evidence to support that *Arabidopsis* initiates early signalling and basal innate defense responses, albeit not sufficiently rapid or effective to prevent SACMV establishment.

#### Phytohormone and signalling networks

In order for plants to adapt to both biotic and abiotic stresses in a cost-efficient manner, cross communication between phytohormone signalling pathways must take place. Signalling pathways may be activated at the same time, depending on the type of pathogen or they may function to act synergistically or antagonistically in order to attempt to mount the most effective defense responses possible [19], [48], [49], [50], [51], [52], [53], [54]. An example of two such pathways working antagonistically was shown by the suppression of the Jasmonic Acid (JA) pathway by salicylic acid (SA) signalling pathway induction following CaLCuV infection in *Arabidopsis* [10]. JA and ET are also known to work synergistically with each other as shown by several studies, including Penninckx et al. 1998 [51]. In contrast to CaLCuV, in our study, SA, JA, and ET appeared to function concomitantly in infected *Arabidopsis* as both up-regulation of PR genes (SA pathway) and defensin (PDF) genes ((JA/ET pathways) (log2 fold or more) was evident (Table S1). Several pathogenesis-related (PR) genes were up-regulated at 14, 24 and 36 dpi. These included, PR1, AT2G14610 (24 dpi, 1.86, and 36 dpi, 3.04), PR5, AT1G75040 (14 dpi, 2.18, 24 dpi, 1.42, and 36 dpi, 1.56), PR4, AT3G04720 (14 dpi, 3.19, 24 dpi, 3.56, and 36 dpi, 2.00), PR-1-like, AT2G19990 (24 dpi, 2.45), and PR protein, AT2G19970 (24 dpi, 2.15), confirming functioning of the SA pathway. Significant induction of JA/ET responsive genes such as PDF 1.2a,b and c (>9 fold up-regulation) and VSP1 (4.78 fold change) (Table 1, 2 and Table S2) were also noted. Ethylene response factor 4 DNA binding/transcriptional repressor (ERF4)(AT3G15210) was significantly down-regulated (−4.64)(Table 2), indicating a possible switching on of transcription of ET signalling. Concomitant functioning of jasmonate and ethylene response pathways have been shown in a previous study to be required for induction of a plant defensin gene in Arabidopsis [51]. *Cauliflower mosaic virus*, a compatible pathogen of Arabidopsis, has been shown to engage three distinct (ET/JA/SA) defense-signalling pathways [55]. PR and PDF transcripts were dominantly prevalent in apical leaves, suggesting that all three pathways, SA, JA and ET, are operational/activated by SACMV in *Arabidopsis* and are acting synergistically with each other, as shown by the induction of marker genes such as PR and PDF (Table S2). However, JA/ET signalling may be favoured over SA pathway since marker genes for JA/ET were more highly induced throughout the study, compared with SA. A basal type of resistance response is ongoing, but is unable to prevent SACMV replication and systemic movement.

Auxin has been shown to be involved in disease susceptibility to viral pathogens [56], [57], [58], [59], for example TMV, where the 126 and 183 kDa replicase disrupts interacting Aux/IAA proteins promoting disease development [56]. In addition, Aux/IAA proteins were also shown to be down-regulated by PPV in *Arabidopsis* (AT5G57420 and AT1G52830) [6]. SA, on the other hand, is able to affect disease susceptibility by repressing the auxin receptor F-box protein TIR1 (Transport Inhibitor response 1, ubiquitin-protein ligase, AT3G62980) causing enhanced resistance [60]. This was not evident in this study as TIR1 was not repressed but up-regulated at 24 dpi (1.52). Furthermore, all auxin-responsive genes identified in our >log2 fold change category were activated by infection (Tables 1, 2), suggesting that, together with evidence of TIR1 activation, symptom and disease progression was allowed to continue in *Arabidopsis*. Indeed, the auxin-responsive protein, AT4G38860 (SAUR-like auxin responsive), was up-regulated at 14 dpi (2.98), 24 dpi (3.06), and 36 dpi (2.45) and IAA29 (AT4G32280) was also induced at 14, 24, and 36 dpi (2.32; 3.27; and 2.57, respectively). It may be advantageous for a geminivirus to regulate this pathway as a means to create a favourable cellular environment for replication in apical leaves.

Brassinosteroids control many aspects of plant growth and development, and are able to induce broad spectrum resistance, but their connection to SA/JA/ET remains to be established [61], [62]. A receptor - like kinase, BAK1 has been shown to interact with receptors that recognize pathogen molecules. BRI1 is one member of a family of leucine-rich receptor-like kinase (LRR-RLK) receptors which interacts with BAK1 upon brassinosteroid perception, initiating the signalling pathway involved in growth - and development related processes [63]. Although the roles of BAK1 in immunity and in brassinosteroid signalling seem to function independently and remain to be elucidated, BRI1 (AT4G39400) was down-regulated in our study at 14 dpi (−1.19), and a BKI1 kinase inhibitor (AT5G42750) was shown to be up-regulated at 24 dpi (1.37) indicating SACMV-induced suppression of the BR1 receptor. This in turn would disrupt brassinosteroid signal transduction as transduction requires heterodimerisation of BRI1 and BAK1 to elicit transcriptional activation of responsive genes. In the same way as C4 of another geminivirus, *Beet curly top virus* (BCTV), may suppress antiviral host defence by disrupting LRR-RLK activity [64], prevention of brassinosteroid-associated signal perception and downstream deactivation of the LRR-RLK BRI1 by SACMV may contribute to failure to activate transcription of resistance-related responsive genes.

#### Signalling and cell-cycle regulation comparison with the bipartite geminivirus, CaLCuV

**Figure 6:**
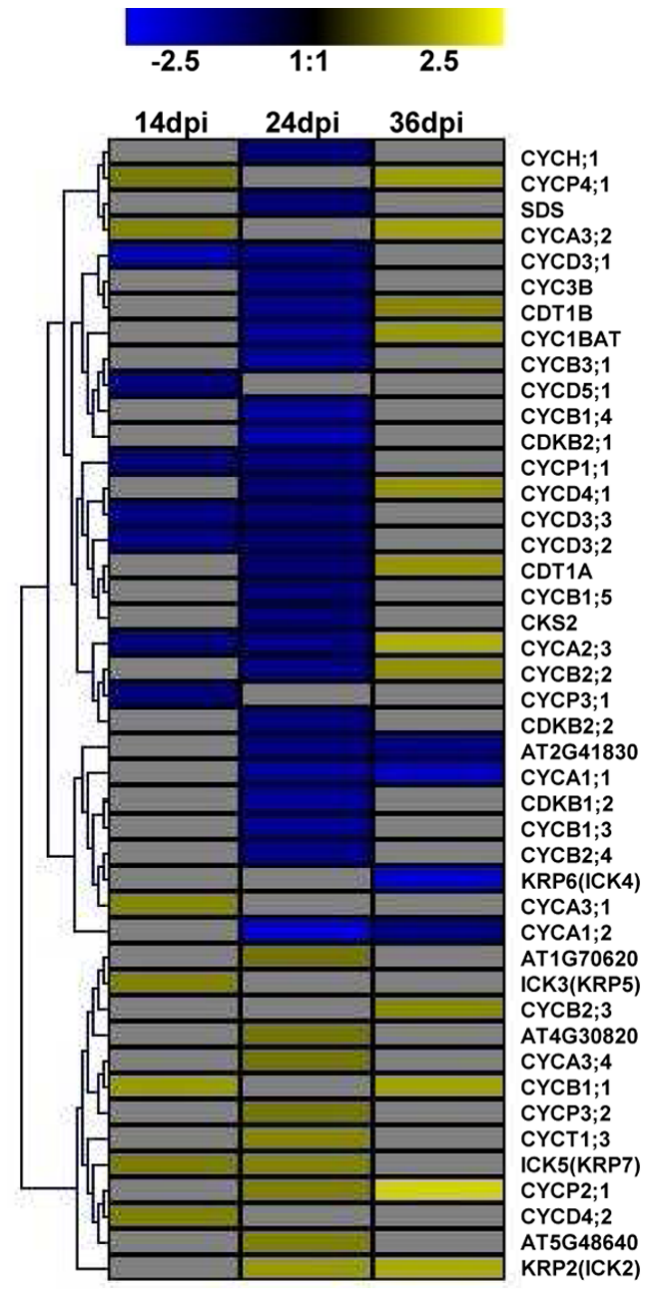
Gene tree heat map of differentially expressed core-cyclin genes in response to SACMV infection. All listed *Arabidopsis* accession numbers refer to cyclin-related genes.

**Figure 7:**
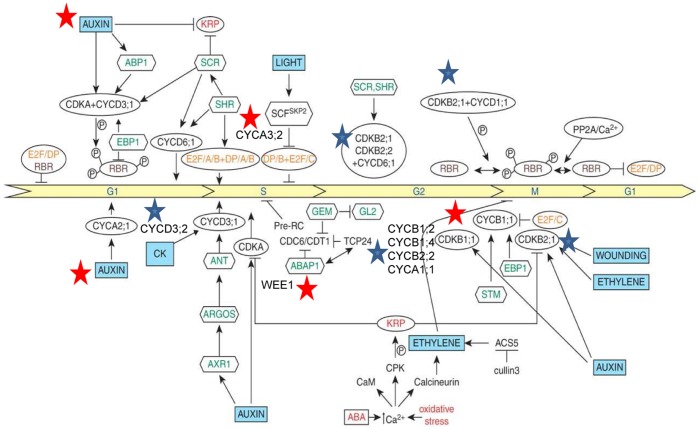
Map of potential links between hormonal signals and cell cycle regulators. Abbreviations: CK, cytokinin; E2F/DP, transcription factors; RBR, retinoblastoma-related protein; P, phospho-protein; CYC, cyclin; CDK, cyclindependent kinase; PP2A, phosphatase; SCR, SCARECROW; SHR, SHORT ROOT; SCF, SKP1+ CULLIN+F-box (SKP2); EBP1, plant homologue of epidermal growth factor-binding protein; SKP2, F-box protein; STM, SHOOT MERISTEMLESS; KRP, CDK inhibitor; CaM, calmodulin; CPK, calmodulin-like domain protein kinase; ABAP1, armadillo BTB Arabidopsis protein 1; TCP24, transcription factor; CDT1, DNA replication-licensing factor; ABP1, auxin binding protein 1; ANT, aintegumenta; ARGOS, auxin-regulated gene in organ size; AXR1, RUB1-activating enzyme; ABA, abscisic acid; GL2, GLABRA (root hair); GEM, GL2 expression regulator; ACS5, 1-aminocyclo-propane-1-carboxil acid synthase [72]. Stars depict SACMV-[ZA:99] involvement in hormone signals and cell cycle regulators. Red stars show up-regulation, while blue stars show down-regulation.

Several core cell-cycle genes were found to be differentially expressed in this study (Figure 6). Functional links between plant signalling hormones (auxin, ethylene, brassinosteroids and cytokinins), and cell-cycle proteins have been established [62], [65], and this is depicted in figure 7. Plant hormones may either directly influence cell-cycle entry and transition or indirectly through developmental regulatory proteins. It has been shown that auxin may stimulate entry into the S-phase, as shown by an increase in histone H4 promoter activity. We believe that SACMV may be responsible for the induction of auxin partly in order to promote S-phase activation. As evidenced by CaLCuV-induced core cell cycle gene transcriptional alterations, geminiviruses manipulate the core cell cycle genes (induce S-phase and G2 genes) in order to provide a replication-enabling environment [10]. A similar finding was observed with SACMV, where 44 of the 61 core cell cycle genes [66] were differentially expressed (Figure 6). We believe this to hold true for SACMV as cyclin genes, such as S-phase CYCA3;2, were induced at both 14 dpi (1.32) and at 36 dpi (1.61). In addition, an auxin-responsive factor protein (AT4G38860) was shown to be up-regulated consistently across time points strongly supporting our hypothesis (Figure 4, Table 1).

CYCB1;1 and CDKB2;1 both promote mitosis and growth in *Arabidopsis*, however opposite effects on expression were noted in both SACMV and CaLCuV studies (Table S6). Down-regulation of CDKB2;1 was noted in both SACMV at 24 dpi (−1.69 fold change) and CalCuV at 12 dpi, while CYCB1;1 was induced by both viruses, and in SACMV-infected Arabidopsis remained induced even at 36 dpi. The SACMV results support the proposal suggested by Ascencio-Ibanez et al [10], that elevated CYCB1;1 leads to sequestering factors necessary for G2 arrest, while reduced CDKB2;1 expression at the G2/M boundary maintains G2 and blocks entry into the M phase, leading to shut down of meristem during infection. In an abiotic stress response study, upon gamma-ray (IR) induction [67], G2/M phase inducers such as CYCB2;1 (and CYCB1;4, CYCB2;2, CYCA1;1) and CDKB1;2, were down-regulated, but CYCB1;1 was induced, similar to biotic stresses (CaCuLV) [10] and SACMV, as mentioned above. G2 to M transition takes place with CDK complexes containing CYCA and CYCB cyclins. WEE1 kinases and inhibitory proteins (CKI’s) phosphorylate CDK complexes in order to keep them in their inactive states. The CKI protein is released by positive phosphorylation by CAK kinase and an unknown protein at the G2 to M boundary, and the kinase is activated [68]. A link in SACMV-infected *Arabidopsis* between CYCB1;1 and auxin is suggested by the observation that the CYCB1;1′s promoter contains an auxin response factor (ARF) binding site [67]. Negative regulators of CDKA;1, namely WEE1, expressed at S-phase, were shown to be up-regulated upon IR induction, most likely to ensure that cell division is delayed from G2 to M [69]. WEE1 (AT1G02970) was also elevated upon SACMV infection, supporting the above-mentioned hypothesis that the G2 phase is maintained by geminiviruses. It is also suggested that, as KRPs (encoding a cyclin-dependent kinase inhibitor) normally function as a negative regulators of cell division [70], induction of KRP2 and KRP5 by SACMV at 24 dpi and 14 dpi, respectively, may contribute to M phase repression. The interaction between phytohormone signalling and cell cycle gene pathways (Figure 7) illustrates that these pathway genes may be co-ordinately suppressed or induced by geminiviruses when required. Here we suggest that SACMV has a concomitant impact on cell-cycle progression and selected hormones that influence the pathways.

Certain features that control the cell-cycle are conserved among eukaryotes in order to ensure mitosis does not begin until DNA replication is completed [68], [71]. Cyclin-dependent kinases bind to the various cyclin types according the phase of the cycle they are entering, and are responsible for transit through control points in cell-regulation. It is the cyclin which determines the specificity and sub-cellular localization, as it is the regulatory component of the complex and can be classified into G1, S and G2-phases [68], [71]. In addition, CDKs are also regulated by interacting proteins and posttranslational modifications (Figure 7) [68]. In general, G1 to S transition phases are controlled by CDK containing D-type cyclins which function to release E2F transcription factors in order for transcription of genes necessary for G1 to S transition to occur. They do this by phosphorylating the retinoblastoma protein (RBR) [10], [68]. It was demonstrated that CaLCuV-infected *Arabidopsis* cells only pass through the early G1 phase since genes such as CYCD1;1 and CYCD3;2 were down-regulated [10]. Differentially expressed core cell cycle genes detected in the SACMV-*Arabidopsis* array were not always picked up in the CaLCuV-*Arabidopsis* hybridization. However a comparison between differentially regulated gene expression between the two geminiviruses (Table S6) showed some similarities. While CYCD1;1 was not detected in the SACMV study, CYCD3;2 was also reduced by SACMV at 14 dpi (−1.38) and at 24 dpi (−1.15), indicating it is likely that geminivirus-infected cells only transit through late G1 [10]. Additionally, late G1 cyclin CYCD4;2 was induced by both SACMV and CaLCuV (Table S6). CaLCuV AC1 binding to RBR causes changes to E2F (E2FA and E2FC) expression by bypassing the G1 phase leading to induction of the endocycle. CYCD3’s normal function is to promote the mitotic cycle and prevent endocycle [10]. Thus, down-regulation of CYCD’s prevent the mitotic cycle from taking place. In addition, genes such as CYCD3;1, CYCD3;2, and CYCD3;3 mutants showed severe symptoms at 12 dpi in CaLCuV suggesting that CaLCuV replicates in endocycling cells. In this study, SACMV infection led to a similar response compared with CaLCuV, as down regulation of CYCD3;1, CYCD3;2 and CYCD3;3 was persistent at 14 and 24 dpi.

The above listed similarities in cell cycle regulation which occur upon biotic stresses such as CalCuV and SACMV infection provided some insight into what is required for geminiviruses to establish a replication-efficient environment, and in addition, similarities shown between abiotic stresses, such as IR induction, confirms that certain cell-cycle regulators are conserved, as previously suggested in other studies.

#### Comparison of data between SACMV and the monopartite geminivirus *Tomato yellow leaf curl virus* (TYLCV)

In a comparative investigation of gene expression changes induced by TYLCV in *Nicotiana benthamiana* [15], we identified 27 common genes with SACMV (Table 3). Many of these genes were shown to have either no effect on infection by TYLCV, or were involved in promotion of earlier infection or in a delay or reduction of infection. The three genes with the highest fold change in SACMV-infected *Arabidopsis* were histone 3 K4-specific methyltransferase (2.38 fold change), which was up-regulated, and two genes which were significantly down-regulated, namely a putative transcriptional activator with NAC domain (−2.16) and a scarecrow-like protein (SCL13) (−2.41). Histone 3 K4-specific methyltransferase and the putative transcriptional activator with NAC domain protein (ATAF1) have been shown to interact with monopartite geminiviral proteins, namely TrAP/C2 and C3, respectively, while the scarecrow-like protein has been found to be a transcription factor, and overexpressed in phloem [72]. Histone 3 K4-specific methyltransferase is located in the chloroplast but its function is not known. A NAC domain protein (SINAC1) was shown to be induced by *Tomato leaf curl virus* (TLCV), interact with the replication enhancer protein of TLCV in tomato, and promote replication [73]. Furthermore, interaction of TMV replicase protein with a NAC domain transcription factor (ATAF2) has also been shown to be associated with suppression of systemic host defences, promoting systemic virus accumulation [74]. In SACMV, down-regulation of ATAF1 at 24 dpi would appear to behave in contradiction to the TMV and TLCV study, and it would be interesting in future to ascertain whether it can bind to SACMV AC2/AC3 proteins.

**Table 3:** Identification of SACMV-induced log2-fold differentially expressed *Arabidopsis* host genes (p<0.05) showing similarities to *Tomato yellow leaf curl Sardinia virus* (TYLCSV) virus in *N. benthamiana* (Lozano-Durán et al, 2011).

*Arabidopsis* Acc no.	SACMV-[ZA:99] Fold Change 14 dpi	SACMV-[ZA:99] Fold Change 24 dpi	SACMV-[ZA:99] Fold Change 36 dpi	Identity	Function	Selection criteria for TYLCV	
				**Group A: no detected infection effects**			
AT2G02560		−1.47		Cullin-associated and neddylation-dissociated (CAND1)	Protein metabolism	TrAP/C2 interaction	
AT1G67630		−1.5		DNA polymerase alpha 2 (POLA2)	DNA metabolism	Cellular process	
AT5G22220		1.19		E2F transcription factor 1 (E2FB)	Transcription	Cellular process	
AT1G21920	1.61	2.38	1.66	Histone 3 K4-specific methyltransferase SET7/9	Unknown	TrAP/C2 interaction	
AT3G44110		−1.19		Homologue to co-chaperone DNAJ-like protein (ATJ3)	Protein folding	C3 interaction	
AT3G25560	−1.31			NSP interacting kinase 2 (NIK2)	Signal transduction	Phloem over-expression	
AT5G03150		−1.47		Putative nucleic acid binding/transcription factor (JDK)	Unknown	TrAP/C2 interaction	
AT1G01720		−2.16		Putative transcriptional activators with NAC domain (ATAF1)	Transcription	C3 interaction	
AT4G17230	−1.56	−2.41		Scarecrow-like protein (SCL13)	Transcription	Phloem over-expression	
AT5G50580		1.24		SUMO activating enzyme (SAE1B)	Protein metabolism	Cellular process	
AT4G24440	1.35	1.26		Transcription factor IIA gamma chain (TFIIA-S)	Transcription	Phloem over-expression	
AT1G19660	−1.41	1.15		Wound inducive gene (F14P1.1)	Stress	C4 interaction	
				**Group B: early infection promoted**			
AT1G09270			−1.41	Importin alpha isoform 4 (IMPA-4)	Transport	CP interaction	
AT1G15380		1.66		Lactoylglutathione lyase (GLO1)	Stress	C3 Interaction	
AT1G47128	1.2	1.55		Dehydration responsive 21 (RD21)	Stress	V2 interaction	
AT5G22000	−1.21			RING-type E3 ubiquitin ligase (RHF2A)	Protein modification	Transactived by TrAP/C2	
AT2G30110	−1.21			Ubiquitin activating enzyme (UBA1)	Protein modification	TrAP/C2 Interaction	
				**Group C: infection delayed, reduced or prevented**			
AT1G51680	1.21	1.4	−2.5	4-coumarate:CoA ligase (AT4CL1)	Metabolism	Phloem over-expression	
AT3G25760	1.28	−1.9	−3.05	Allene oxide cyclase (AOC1)	Metabolism	Phloem over-expression	
AT5G61430	1.36	1.61		Geminivirus Rep A-binding (GRAB2)	Transcription	Rep interaction	
AT2G26560	1.61			Patatin-like protein 2 (PLP2)	Stress	Phloem over-expression	
AT1G09840		−1.16		Shaggy-related kinase kappa (SK4-1/SKK)	Protein modification	C4 interaction	
AT5G08590		1.19		SKP1-like 2 (ASK2)	Protein modification	Transactived by TrAP/C2	

Genes such as NSI, GRAB2, and RPA32 were also shown to modify TYLCSV infection in *N. benthamiana* (Table 3) [15]. In SACMV-infected *Arabidopsis*, GRAB2 was up-regulated at 14 dpi (1.36) and at 24 dpi (1.61), respectively. GRAB2 is a Rep A binding protein whose exact role in replication initiation is unclear. An increase in expression was shown to cause inhibition of replication of the monopartite geminivirus, *Wheat dwarf virus* (WDV) [75], whereas in contrast, down-regulation of GRAB2 caused inhibition of TYLCSV infection indicating that GRAB2 is required for complete infectivity but that the appropriate expression levels are critical [15]. According to the TYLCSV study by Lozano-Durán et al. 2011 [15], 8 of the 18 differentially expressed genes involved in protein modifications, were associated with ubiquitination, acetylation, protein folding, phosphorylation and rubylation, four of which were involved in ubiquitination (UBA1, RHF2A, ASK2, and CSN3). UBA1 was found to be down-regulated by SACMV at 24 dpi (−1.21). This gene is involved in many levels of plant defense, one of which is virus resistance. Down-regulation of this gene by both a monopartite and bipartite geminivirus, TYLCSV and SACMV, respectively, favours the proposal that a geminiviral protein interaction, C2 protein in the case of TYLCSV, inhibits UBA1-mediated ubiquitination of possible viral proteins or host protein(s) linked to a resistance-associated response, which would favour progression of infection. Silencing of UBA1 resulted in early TYLCSV infection, supporting this theory. RFH2A was also silenced by TYLCSV, prolonging virus infection, and this gene was also found to be repressed by SACMV at 14 dpi (−1.21) (Table 3) confirming its likely role in sustaining virus infection. It has also been suggested that this gene may be involved in counteracting plant defense, as it was up-regulated by CaLCuV in *Arabidopsis* at 12 dpi [10]. Genes identified in biotic stress responses (RD21, GLO1, and PLP2) upon TYLCSV infection were also induced by SACMV at 14 dpi and/or 24 dpi, demonstrating that geminiviruses, in addition to RNA plant viruses in general [3], initiate basal innate plant defense responses, and that this is not unique to a particular group of pathogens. AOC1, involved in JA biosynthesis was differentially expressed at all 3 time points upon SACMV infection [up-regulated at 14 dpi (1.28) and down-regulated at 24 dpi (−1.87) and 36 dpi (−3.05)], but up-regulation early in infection (14 dpi) suggests an early non-specific JA-associated broad defense host response, as discussed previously. In contrast, AOC1 was reduced by CaLCuV infection, correlating with its suppression of the JA pathway and the induction of the SA pathway.

#### Selected genes of interest with more than log2 fold expression changes

Plant defensins are cationic antimicrobial peptides, belonging to classes four and five, and are involved in plant innate immunity [76]. The *Arabidopsis* defensins are divided into three families. PDF1-3 [77] and expression of defensins are highly regulated, usually linked to the ET and JA pathways [51]. For example, PDF1.2a (AT5G44420) which is a low molecular weight cysteine-rich protein, is highly responsive to ET and JA, and is involved in JA- and ET-.dependent systemic resistance. This PDF is not responsive to salicylic acid and is located in the cell wall and extracellular region. PDF1.2b (AT2G26020) and PDF1.2c (AT5G44430) encode for pathogenesis-related (PR) proteins involved in the ET-mediated signalling pathway, and are also cell wall and extracellularly located. PDF1.3 is a PR-protein which is involved in innate defense responses [77]. PDF1.2a, b, and c, and PDF1.3 represented some of the most highly up-regulated genes (6.14–15.82 fold changes) across all time points in this study (Tables 1 and 2). Transcription factors ERF1 and ORA59 form part of the APETALA2/ETHYLENE RESPONSE FACTOR (AP2/ERF) superfamily. The AP2/ERF domains bind to a GCC promoter box of stress-responsive genes, and can act as either activators or repressors of stress responsive genes [54], [59], [78]. AP2 domain-containing transcription factors were down-regulated across all time points at a log 2 fold cut-off (Figure 4, Table 1). In an abiotic stress response study conducted by Brini et al 2011 [79], down-regulation of AP2 domain-containing transcription factors and up-regulation of plant defensin genes such as PDF1.2 was evident, illustrating a common trend in expression patterns to both abiotic and biotic stress responses. Plant defensin genes were highly up-regulated in our study suggesting that JA/ET signalling pathways were acting synergistically or concomitantly, leading to up-regulation of these genes in response to SACMV.

Toll-interleuken-1-receptor/nucleotide binding site/leucine rich repeat (TIR-NBS-LRR) is a disease resistance protein which confers specific resistance to viral diseases. This was up-regulated (10.84) in *Arabidopsis* protoplasts by the RNA virus, *Plum pox virus* (PPV) [6], but was down-regulated by SACMV in *Arabidopsis* leaves. Repressed TIR-NBS-LRR disease resistance proteins for SACMV infection in *Arabidopsis* were as follows:- AT5G41740 (−2.76 (14 dpi), −2.47 (24 dpi)), AT3G44630 (−2.08, 24 dpi), AT4G19520 (−2.30 (14 dpi), −2.24 (24 dpi)), AT5G41550, −2.48 (24 dpi), AT5G18360 (−2.32, 24 dpi), AT5G22690 (−2.98, 24 dpi), AT5G58120 (−2.03, 24 dpi), AT1G56510 (−2.89, 24 dpi), and AT1G56540 (−2.02, 24 dpi)]. TIR-NBS-LRR protein down-regulation supports a model that SACMV suppresses these disease resistance proteins in order to allow for replication and spread.

Little is known about cell-to-cell movement of geminiviruses, and we were keen to identify putative host proteins known to play a role in RNA virus movement [80]. ß-1,3-glucanase (BGL2) (AT3G57260), BGLU46 and BGL1 (Table 2) were found to be up-regulated by SACMV at all three time points, especially at 14 dpi (3.01) [24 dpi (1.73), and 36 dpi (1.36)], with 14 dpi showing the highest expression. Callose deposition/removal and ß-1,3-glucanase activity have been associated with plasmadesmatal (Pd) gate modifications [81], [82]. Degradation of callose by ß-1,3-glucanases increases the Pd size exclusion limit (SEL), and has been implicated in facilitating cell-to-cell movement of RNA viruses [81], [82]. RNA viruses (TVCV, ORMV, PVX, CMV, and TuMV) all demonstrated elevated ß-1,3-glucanase activity at 2,4,5 DAI (days after infection), increasing exponentially over the time course of infection [3]. Another interesting gene, 4CL1, is responsible for channelling carbon flow in the phenylpropanoid metabolic pathway. It appears to be involved in cell wall modification as silencing of this gene caused increased cellulose and decreased lignin in general [83], [84]. 4CL1 was shown to be up-regulated at 14 dpi (1.21) and 24 dpi (1.40), and significantly down-regulated at 36 dpi (−2.50) by SACMV, indicating a possible synergistic role, along with ß-1,3-glucanase, in SACMV cell-to-cell movement via cell wall modifications. Up-regulation of ß-1,3-glucanase and callose breakdown, along with decreased lignin production in this SACMV-*Arabidopsis* interaction, strongly supports involvement in cell wall modification at the Pd location in facilitating geminivirus cell-to-cell movement, and may argue for a cell-wall “loosening” associated mechanism and Pd gate expansion model as a general conserved plant response to many RNA and DNA virus infections.

Two important protein families of interest in virus-host interactions are those belonging to the proteosome-related and heat shock protein (HSPs) associated pathways [3], [4], [6], [85], [86], [87], [88]. In *Plum pox virus* (PPV) infection study [6], genes associated with the 26S proteasome were found to be highly significantly (Q <0.05), up-regulated, one of which being AAA-ATPAse. The 26S proteosome functions to control degradation of regulatory target proteins such as virus-encoded movement proteins, suggesting an involvement in resistance [6]. In this study, AAA type ATPase family protein (AT2G18193) was shown to be highly up-regulated across three time-points [4.25(14 dpi), 4.57(24 dpi), and 3.51(36 dpi)] (Table 1). This suggests that a basal resistance may be activated but is not sufficient enough to counteract SACMV attack as an increase in virus titre across the time line was evident, resulting in a susceptible interaction (Figure 1C, and Figure 4,Table 1).

HSP’s are involved in a wide range of functions in both abiotic and biotic cellular stress and in plant growth and development, and are controlled at the transcriptional level [3], [4], [87], [88], [89], [90]. In many plant studies with RNA viruses, HSP’s are shown to be up-regulated as a general stress response upon virus attack [3], [6]. Little is known about HSP’s associated with host responses to DNA viruses, but mention was made to induction of HSP70 in response to the geminivirus, *Beet curly top virus* [91]. In this study, we were surprised to observe that many HSP’s were down-regulated at a log2 fold cut-off (Table S2) and several small class III heat shock proteins (HSP17.4-CIII); HSP17.8-Cl) and HSP17.6A-Cl were also found to be highly repressed across all time points (Table 1). *Arabidopsis* cytosolic HSP17.6A was shown to be a chaperone protein, induced by heat and osmotic stress [92], and HSP17.8 functions as an AKR2A cofactor in targeting the chloroplast outer membrane proteins in Arabidopsis [93]. Since many HSPs are up-regulated by abiotic and biotic stress, opposite findings in our study suggest multiple roles for HSPs in both general and geminivirus-specific stress responses and possibly virus replication. Li et al., 2011 [89] recently identified a heat shock protein 70 (HSP70) which may play multiple roles in virus replication of influenza A, such as interaction with the influenza virus ribonucleprotein (RNP) complex, which is involved in negative regulation of influenza A transcription and replication in infected cells. HSP70 may also assist with subcellular localization and membrane insertion of viral replication proteins and assembly of viral replicase [87], [89].

In *Arabidopsis,* heat shock proteins were induced by five RNA viruses (ORMV,TVCV, CMV, Potato virus X and TuMV) and by SYMV and INSV (negative-strand RNA viruses) in *N. benthamiana* [3]. Of the HSP’s (HSP70 and HSP90) showing chaperone activity in the Agudelo-Romero et al. 2008 TEV study [71], one of the HSP’s (HSP70,AT3G12580) in particular was also identified in our SACMV-*Arabidopsis* study, but showed opposite expression. HSP70 (AT3G12580) was up-regulated by TEV and down-regulated by SACMV (−1.98 at 14dpi, and −2.36 at 24dpi). This finding, again supports the earlier suggestion that HSP70 may play different roles at different times in virus-infected plants and that differential regulation of HSP’s is not always a general stress response but may be specifically targeted by a geminivirus at a particular stage of infection for its own benefit, for example replication or cell-to-cell movement, where HSP70 family chaperones may well be exploited in general folding of movement protein-nucleic acid complexes [80], or regulation of host defenses directly or indirectly through interactions with J-domain proteins [94]. It has been suggested that one of the replicase, movement or 16-KDa proteins encoded by RNA1 of *Pea early browning virus* (PEBV) was possibly the elicitor for induction of HSP70 expression [91]. If this is the case, we suggest that if a movement protein is capable of eliciting HSP’s (in particular HSP70) then it is also capable of suppressing HSP expression which is evident with significantly (p<0.05) down-regulated HSP’s identified at a log2 fold cut-off in SACMV infection. Down-regulation of HSPs was also maintained across the 36 day infection period. We think it not unreasonable to argue that down regulation may be mediated by SACMV in order to suppress innate immune responses, and redirect cellular pathways for its own replication and movement, and also suggest that some geminiviruses may not have an absolute requirement for heat shock for infection progression.

### Conclusions

In conclusion, the large number of genes unveiled in this study provided valuable insight into the little that is known about geminivirus-host interactions. The GO results in this study are consistent with the hypothesis that plant virus stress leads to a transition from normal host growth processes to altered metabolic pathways geared for defense responses. Both similarities and differences were identified between SACMV and the geminiviruses, CaLCuV in *Arabidopsis* and TYLCV in *N. benthamiana*, and other RNA viruses, identifying general as well as virus-specific responses in a host. Importantly, we also demonstrate that different altered gene profiles occur at early, middle and late infection stages, and that a limited number of genes are differentially expressed across the entire infection period. Differences between geminiviruses in the same host, *Arabidopsis*, demonstrate that many host responses in a compatible interaction are geminivirus-specific, and differences in expression patterns may in part be a reflection of different adaptation and evolutionary histories of the viruses and their hosts. This is supported by the comparative microarray study of Arabidopsis, where, while some overlap in altered expression between different viruses in this host occurred, virus-host interactions were essentially unique [34]. It is evident that many host defense layers exist which viruses need to overcome in order to establish successful infection. The suppressive nature of SACMV on many host genes revealed that in a compatible interaction, basal defences are induced but are not capable of inhibiting viral replication and spread, as demonstrated by the progressive increase in symptom severity, virus titre and high number of repressed genes over the infection period. Identifying gene interactions in signalling pathways is a step closer toward identifying master transcription factors controlling these networks. A more systems biology approach will be adopted in further studies to connect these networks. Host-responsive genes may also be grouped or clustered based on their co-expression pattern or chromosomal location, and this also needs to be investigated. Functional testing of candidate genes and transcription factors through a reverse genetics approach, RNA silencing, VIGS and miRNA studies, will also be the next step in expanding on our knowledge of geminivirus-host interactions.

## Supporting Information

Table S1 **Log2-fold differentially expressed genes (p<0.05) after normalization.**
(XLSX)

Table S2 **Selected differentially expressed genes showing log2-fold change and adjusted P-Values (p<0.05).**
(XLSX)

Table S3 **A comparison of differentially expressed genes between SACMV and eleven plant viruses in Arabidopsis.**
(XLSX)

Table S4 **Comparative expression data between SACMV and CaLCuV infected Arabidopsis.**
(XLSX)

Table S5 **Comparative expression data between SACMV, CaLCuV and 11 RNA viruses in Arabidopsis.**
(XLSX)

Table S6 **Log2 fold changes of differentially expressed selected core cell cycle genes of SACMV and CaLCuV.**
(XLS)

## References

[1] Dardick C (2007) Comparative expression profiling of Nicotiana benthamiana leaves systemically infected with three fruit tree viruses. Mol Plant Microbe Interact 20(8): 1004–1017. 17722703 10.1094/MPMI-20-8-1004

[2] Trinks D, Rajeswaran R, Shivaprasad PV, Akbergenov R, Oakeley EJ, et al (2005) Suppression of RNA silencing by a geminivirus nuclear protein, AC2, correlates with transactivation of host genes. J Virol 79(4): 2517–2527. 15681452 10.1128/JVI.79.4.2517-2527.2005 PMC546592

[3] Whitham SA, Quan S, Chang HS, Cooper B, Estes B, et al (2003) Diverse RNA viruses elicit the expression of common sets of genes in susceptible Arabidopsis thaliana plants. Plant J 33(2): 271–283. 12535341 10.1046/j.1365-313x.2003.01625.x

[4] Whitham SA, Yang C, Goodin MM (2006) Global impact: elucidating plant responses to viral infection. Mol Plant Microbe Interact 19(11): 1207–1215. 17073303 10.1094/MPMI-19-1207

[5] Agudelo-Romero P, Carbonell P, Perez-Amador MA, Elena SF (2008) Virus adaptation by manipulation of host’s gene expression. PLoS One 3(6): e2397.a. 18545680 10.1371/journal.pone.0002397 PMC2398778

[6] Babu M, Griffiths JS, Huang TS, Wang A (2008) Altered gene expression changes in Arabidopsis leaf tissues and protoplasts in response to Plum pox virus infection. BMC Genomics 9: 325.a. 18613973 10.1186/1471-2164-9-325 PMC2478689

[7] Babu M, Gagarinova AG, Brandle JE, Wang A (2008) Association of the transcriptional response of soybean plants with soybean mosaic virus systemic infection. J Gen Virol 89(Pt 4): 1069–1080.b. 10.1099/vir.0.83531-0 18343851

[8] Nagar S, Pedersen TJ, Carrick KM, Hanley-Bowdoin L, Robertson D (1995) A geminivirus induces expression of a host DNA synthesis protein in terminally differentiated plant cells. Plant Cell 7(6): 705–719. 7647562 10.1105/tpc.7.6.705 PMC160820

[9] Havelda Z, Hornyik C, Crescenzi A, Burgyan J (2003) In situ characterization of Cymbidium Ringspot Tombusvirus infection-induced posttranscriptional gene silencing in *Nicotiana benthamiana* . J Virol 77(10): 6082–6086. 12719602 10.1128/JVI.77.10.6082-6086.2003 PMC154021

[10] Ascencio-Ibanez JT, Sozzani R, Lee TJ, Chu TM, Wolfinger RD, et al (2008) Global analysis of Arabidopsis gene expression uncovers a complex array of changes impacting pathogen response and cell cycle during geminivirus infection. Plant Physiol 148(1): 436–454. 18650403 10.1104/pp.108.121038 PMC2528102

[11] Owens RA, Tech KB, Shao JY, Sano T, Baker CJ (2012) Global analysis of tomato gene expression during Potato spindle tuber viroid infection reveals a complex array of changes affecting hormone signaling. Mol Plant Microbe Interact 25(4): 582–598. 22217247 10.1094/MPMI-09-11-0258

[12] Elena SF, Carrera J, Rodrigo G (2011) A systems biology approach to the evolution of plant-virus interactions. Curr Opin Plant Biol 14(4): 372–377. 21458360 10.1016/j.pbi.2011.03.013

[13] Maule A, Leh V, Lederer C (2002) The dialogue between viruses and hosts in compatible interactions. Curr Opin Plant Biol 5(4): 279–284. 12179959 10.1016/s1369-5266(02)00272-8

[14] Carrington JC, Whitham SA (1998) Viral invasion and host defense: strategies and counter-strategies. Curr Opin Plant Biol 1(4): 336–341. 10066606 10.1016/1369-5266(88)80056-6

[15] Lozano-Duran R, Rosas-Diaz T, Luna AP, Bejarano ER (2011) Identification of host genes involved in geminivirus infection using a reverse genetics approach. PLoS One 6(7): e22383. 21818318 10.1371/journal.pone.0022383 PMC3144222

[16] Pallas V, Garcia JA (2011) How do plant viruses induce disease? Interactions and interference with host components. J Gen Virol 92(Pt 12): 2691–2705. 10.1099/vir.0.034603-0 21900418

[17] Ballare CL (2011) Jasmonate-induced defenses: a tale of intelligence, collaborators and rascals. Trends Plant Sci 16(5): 249–257. 21216178 10.1016/j.tplants.2010.12.001

[18] Koornneef A, Pieterse CM (2008) Cross talk in defense signaling. Plant Physiol 146(3): 839–844. 18316638 10.1104/pp.107.112029 PMC2259093

[19] Pieterse CM, Leon-Reyes A, Van der Ent S, Van Wees SC (2009) Networking by small-molecule hormones in plant immunity. Nat Chem Biol 5(5): 308–316. 19377457 10.1038/nchembio.164

[20] Berrie LC, Rybicki EP, Rey ME (2001) Complete nucleotide sequence and host range of *South African cassava mosaic virus*: further evidence for recombination amongst begomoviruses. J Gen Virol 82(Pt 1): 53–58. 10.1099/0022-1317-82-1-53 11125158

[21] Harrison BD, Robinson DJ (2002) Green shoots of geminivirology. Physiological and Molecular Plant Pathology 60: 215–218.

[22] Gorovits R, Akad F, Beery H, Vidavsky F, Mahadav A, et al (2007) Expression of stress-response proteins upon whitefly-mediated inoculation of Tomato yellow leaf curl virus in susceptible and resistant tomato plants. Mol Plant Microbe Interact 20(11): 1376–1383. 17977149 10.1094/MPMI-20-11-1376

[23] Gafni Y, Epel BL (2002) The role of host and viral proteins in intra- and inter-cellular trafficking of geminiviruses. Physiological and Molecular Plant Pathology 60: 231–241.

[24] Fontes EP, Santos AA, Luz DF, Waclawovsky AJ, Chory J (2004) The geminivirus nuclear shuttle protein is a virulence factor that suppresses transmembrane receptor kinase activity. Genes Dev 18(20): 2545–2556. 15489295 10.1101/gad.1245904 PMC529541

[25] Jeske H (2009) Geminiviruses. Curr Top Microbiol Immunol 331: 185–226. 19230564 10.1007/978-3-540-70972-5_11

[26] Mariano AC, Andrade MO, Santos AA, Carolino SMB, Oliveira ML, et al (2004) Identification of a novel receptor-like protein kinase that interacts with a geminivirus nuclear shuttle protein. Virology 318: 24–3. 14972531 10.1016/j.virol.2003.09.038

[27] Mills-Lujan K, Deom CM (2010) Geminivirus C4 protein alters Arabidopsis development. Protoplasma 239(1–4): 95–110. 20091067 10.1007/s00709-009-0086-z

[28] Gutierrez C (2000) DNA replication and cell cycle in plants: learning from geminiviruses. Embo J 19(5): 792–799. 10698921 10.1093/emboj/19.5.792 PMC305619

[29] Gutierrez C (2002) Strategies for geminivirus DNA replication and cell cycle interference. Physiological and Molecular Plant Pathology 60: 219–230.

[30] Hanley-Bowdoin L, Settlage SB, Robertson D (2004) Reprogramming plant gene expression: a prerequisite to geminivirus DNA replication. Mol Plant Pathol 5(2): 149–156. 20565592 10.1111/j.1364-3703.2004.00214.x

[31] Ma S, Gong Q, Bohnert HJ (2007) An Arabidopsis gene network based on the graphical Gaussian model. Genome Res 17(11): 1614–1625. 17921353 10.1101/gr.6911207 PMC2045144

[32] Geisler-Lee J, O’Toole N, Ammar R, Provart NJ, Millar AH, et al (2007) A predicted interactome for Arabidopsis. Plant Physiol 145(2): 317–329. 17675552 10.1104/pp.107.103465 PMC2048726

[33] Busch W, Lohmann JU (2007) Profiling a plant: expression analysis in Arabidopsis. Curr Opin Plant Biol 10(2): 136–141. 17291825 10.1016/j.pbi.2007.01.002

[34] Postinova OA, Nemchinov LG (2012) Comparative analysis of microarray data in Arabidopsis transcriptome during compatible interactions with plant viruses. Virology J 9: 101. 22643110 10.1186/1743-422X-9-101 PMC3430556

[35] Koornneef M, Meinke D (2010) The development of Arabidopsis as a model plant. Plant J 61(6): 909–921. 20409266 10.1111/j.1365-313X.2009.04086.x

[36] Fregene M, Matsumura H, Akano A, Dixon A, Terauchi R (2004) Serial analysis of gene expression (SAGE) of host-plant resistance to the cassava mosaic disease (CMD). Plant Mol Biol 56(4): 563–571. 15630620 10.1007/s11103-004-3477-8

[37] Edgar R, Domrachev M, Lash AE (2002) Gene Expression Omnibus: NCBI gene expression and hybridization array data repository. Nucleic Acids Res 30(1): 207–210. 11752295 10.1093/nar/30.1.207 PMC99122

[38] Barrett T, Troup DB, Wilhite SE, Ledoux P, Rudnev D et al.. (2007) NCBI GEO: mining tens of millions of expression profiles–database and tools update. Nucleic Acids Res 35(Database issue): D760–765. 10.1093/nar/gkl887 PMC1669752 17099226

[39] Doyle JJ, Doyle JL (1987) A rapid isolation procedure for small quantities of fresh leaf tissue.Phytochemical Bulletin. 19: 11–15.

[40] Petti C, Wendt T, Meade C, Mullins E (2009) Evidence of genotype dependency within *Agrobacterium tumefaciens* in relation to the integration of vector backbone sequence in transgenic *Phytophthora infestans*-tolerant potato. J Biosci Bioeng 107(3): 301–306. 19269597 10.1016/j.jbiosc.2008.11.012

[41] Chomczynski P, Sacchi N (1987) Single-step method of RNA isolation by acid guanidinium thiocyanate-phenol-chloroform extraction. Anal Biochem 162(1): 156–159. 2440339 10.1006/abio.1987.9999

[42] Andersen CL, Jensen JL, Orntoft TF (2004) Normalization of real-time quantitative reverse transcription-PCR data: a model-based variance estimation approach to identify genes suited for normalization, applied to bladder and colon cancer data sets. Cancer Res 64(15): 5245–5250. 15289330 10.1158/0008-5472.CAN-04-0496

[43] Veena, Jiang H, Doerge RW, Gelvin SB (2003) Transfer of T-DNA and Vir proteins to plant cells by *Agrobacterium tumefaciens* induces expression of host genes involved in mediating transformation and suppresses host defense gene expression. Plant J 35(2): 219–236. 12848827 10.1046/j.1365-313x.2003.01796.x

[44] Smyth GK (2004) Linear models and empirical Bayes methods for assessing differential expression in microarray experiments. Statistical Applications in Genetics and Molecular Biology Volume 3, Issue 1, Article 3. 10.2202/1544-6115.1027 16646809

[45] Fisher RA (1970) Statistical Methods for Research Workers. 14th edition, Hafner Publishing, 1970, p. 96.

[46] O’Donnell PJ, Schmelz EA, Moussatche P, Lund ST, Jones JB, et al (2003) Susceptible to intolerance - a range of hormonal actions in a susceptible Arabidopsis pathogen response. Plant J 33(2): 245–257. 12535339 10.1046/j.1365-313x.2003.01619.x

[47] Golem S, Culver JN (2003) Tobacco mosaic virus induced alterations in the gene expression profile of Arabidopsis thaliana. Mol Plant Microbe Interact 16(8): 681–688. 12906112 10.1094/MPMI.2003.16.8.681

[48] Baena-Gonzalez E (2010) Energy signaling in the regulation of gene expression during stress. Mol Plant 3(2): 300–313. 20080814 10.1093/mp/ssp113

[49] Cheong YH, Chang HS, Gupta R, Wang X, Zhu T, et al (2002) Transcriptional profiling reveals novel interactions between wounding, pathogen, abiotic stress, and hormonal responses in Arabidopsis. Plant Physiol 129(2): 661–677. 12068110 10.1104/pp.002857 PMC161692

[50] Llorente F, Muskett P, Sanchez-Vallet A, Lopez G, Ramos B, et al (2008) Repression of the auxin response pathway increases Arabidopsis susceptibility to necrotrophic fungi. Mol Plant 1(3): 496–509. 19825556 10.1093/mp/ssn025

[51] Penninckx IA, Thomma BP, Buchala A, Metraux JP, Broekaert WF (1998) Concomitant activation of jasmonate and ethylene response pathways is required for induction of a plant defensin gene in Arabidopsis. Plant Cell 10(12): 2103–2113. 9836748 10.1105/tpc.10.12.2103 PMC143966

[52] Ballare CL (2011) Jasmonate-induced defenses: a tale of intelligence, collaborators and rascals. Trends Plant Sci 16(5): 249–257. 21216178 10.1016/j.tplants.2010.12.001

[53] Marathe R, Guan Z, Anandalakshmi R, Zhao H, Dinesh-Kumar SP (2004) Study of Arabidopsis thaliana resistome in response to cucumber mosaic virus infection using whole genome microarray. Plant Mol Biol 55(4): 501–520. 15604696 10.1007/s11103-004-0439-0

[54] Guo H, Ecker JR (2004) The ethylene signaling pathway: new insights. Curr Opin Plant Biol 7(1): 40–49. 14732440 10.1016/j.pbi.2003.11.011

[55] Love AJ, Yun BW, Laval V, Loake GJ, Milner JJ (2005) Cauliflower mosaic virus, a compatible pathogen of Arabidopsis, engages three distinct defense-signaling pathways and activates rapid systemic generation of reactive oxygen species. Plant Physiol 139(2): 935–948. 16169957 10.1104/pp.105.066803 PMC1256007

[56] Padmanabhan MS, Shiferaw H, Culver JN (2006) The Tobacco mosaic virus replicase protein disrupts the localization and function of interacting Aux/IAA proteins. Mol Plant Microbe Interact 19(8): 864–873. 16903352 10.1094/MPMI-19-0864

[57] Culver JN, Padmanabhan MS (2007) Virus-induced disease: altering host physiology one interaction at a time. Annu Rev Phytopathol 45: 221–243. 17417941 10.1146/annurev.phyto.45.062806.094422

[58] Robert-Seilaniantz A, Navarro L, Bari R, Jones JD (2007) Pathological hormone imbalances. Curr Opin Plant Biol 10(4): 372–379. 17646123 10.1016/j.pbi.2007.06.003

[59] Spaepen S, Vanderleyden J (2010) Auxin and plant-microbe interactions. Cold Spring Harb Perspect Biol 3(4). 10.1101/cshperspect.a001438 PMC3062209 21084388

[60] Dharmasiri N, Dharmasiri S, Estelle M (2005) The F-box protein TIR1 is an auxin receptor. Nature 435(7041): 441–445. 15917797 10.1038/nature03543

[61] Nakashita H, Yasuda M, Nitta T, Asami T, Fujioka S, et al (2003) Brassinosteroid functions in a broad range of disease resistance in tobacco and rice. Plant J 33(5): 887–898. 12609030 10.1046/j.1365-313x.2003.01675.x

[62] Bari R, Jones JD (2009) Role of plant hormones in plant defence responses. Plant Mol Biol 69(4): 473–488. 19083153 10.1007/s11103-008-9435-0

[63] Li J, Wen J, Lease KA, Doke JT, Tax FE, et al (2002) BAK1, an Arabidopsis LRR receptor-like protein kinase, interacts with BRI1 and modulates brassinosteroid signaling. Cell 110(2): 213–222. 12150929 10.1016/s0092-8674(02)00812-7

[64] Piroux N, Saunders K, Page A, Stanley J (2007) Geminivirus pathogenicity protein C4 interacts with Arabidopsis thaliana shaggy-related protein kinase AtSKeta, a component of the brassinosteroid signalling pathway. Virology 362(2): 428–440. 17280695 10.1016/j.virol.2006.12.034

[65] Dudits D, Abraham E, Miskolczi P, Ayaydin F, Bilgin M, et al (2007) Cell-cycle control as a target for calcium, hormonal and developmental signals: the role of phosphorylation in the retinoblastoma-centred pathway. Ann Bot 107(7): 1193–1202. 10.1093/aob/mcr038 PMC3091804 21441245

[66] Vandepoele K, Raes J, De Veylder L, Rouze P, Rombauts S, et al (2002) Genome-wide analysis of core cell cycle genes in Arabidopsis. Plant Cell 14(4): 903–916. 11971144 10.1105/tpc.010445 PMC150691

[67] Ricaud L, Proux C, Renou JP, Pichon O, Fochesato S, et al (2007) ATM-mediated transcriptional and developmental responses to gamma-rays in Arabidopsis. PLoS One 2(5): e430. 17487278 10.1371/journal.pone.0000430 PMC1855986

[68] Andrietta MH, Eloy NB, Hermerly AS, Ferreira PCG (2001) Identification of sugarcane cDNAs encoding components of cell cycle machinery. Gen Mol Bio 24(1–4): 61–68.

[69] De Schutter K, Joubes J, Cools T, Verkest A, Corellou F, et al (2007) Arabidopsis WEE1 Kinase Controls Cell Cycle Arrest in Response to Activation of the DNA integrity Checkpoint. Plant Cell 19: 211–225. 17209125 10.1105/tpc.106.045047 PMC1820959

[70] Agudelo-Romero P, Carbonell P, de la Iglesia F, Carrera J, Rodrigo G, et al (2008) Changes in the gene expression profile of Arabidopsis thaliana after infection with *Tobacco etch virus* . Virol J 5: 92.b. 18684336 10.1186/1743-422X-5-92 PMC2518140

[71] Sorrell DA, Menges M, Healy JM, Deveaux Y, Amano C, et al (2001) Cell cycle regulation of cyclin-dependent kinases in tobacco cultivar Bright Yellow-2 cells. Plant Physiol 126(3): 1214–1223. 11457971 10.1104/pp.126.3.1214 PMC116477

[72] Vilaine F, Palauqui J-C, Amselem J, Kusiak C, Lemoine R, et al (2003) Towards deciphering phloem: a transcriptome analysis of the phloem of *Apium graveolens* . Plant J 36: 67–81. 12974812 10.1046/j.1365-313x.2003.01855.x

[73] Selth L, Dogra SC, Rasheed MS, Healy H, Randles JW, et al (2005) NAC Domain Protein Interacts with *Tomato leaf curl virus* Replication Accessory Protein and Enhances Viral Replication. Plant Cell 17: 311–325. 15608335 10.1105/tpc.104.027235 PMC544507

[74] Wang X, Sameer P, Goregaoker P, Culver JN (2009) Interaction of the *Tobacco Mosaic Virus* Replicase Protein with a NAC Domain Transcription Factor is Associated with the Suppression of Systemic Host Defenses. J of Virol 83: 9720–9730. 19625399 10.1128/JVI.00941-09 PMC2748025

[75] Xie Q, Sanz-Burgos AP, Guo H, Garcia JA, Gutierrez C (1999) GRAB proteins, novel members of the NAC domain family, isolated by their interaction with a geminivirus protein. Plant Mol Biol 39(4): 647–656. 10350080 10.1023/a:1006138221874

[76] Broekaert WF, Terras FRG, Cammue BPA, Osborn RW (1995) Plant defensins: novel antimicrobial peptides as components of the host defense system. Plant Physiol 108: 1353–1358. 7659744 10.1104/pp.108.4.1353 PMC157512

[77] Thomma BPHJ, Cammue BPA, Thevissen K (2002) Plant defensins. Planta 216: 193–202. 12447532 10.1007/s00425-002-0902-6

[78] Mishra A, Godavarthi SK, Maheshwari M, Goswami A, Jana NR (2009) The ubiquitin ligase E6-AP is induced and recruited to aggresomes in response to proteasome inhibition and may be involved in the ubiquitination of Hsp70-bound misfolded proteins. J Biol Chem 284(16): 10537–10545. 19233847 10.1074/jbc.M806804200 PMC2667740

[79] Brini F, Yamamoto A, Jlaiel L, Takeda S, Hobo T, et al (2011) Pleiotropic effects of the wheat dehydrin DHN-5 on stress responses in Arabidopsis. Plant Cell Physiol 52(4): 676–688. 21421569 10.1093/pcp/pcr030

[80] Boevink P, Oparka KJ (2005) Virus-host interactions during movement processes. Plant Physiol 138(4): 1815–1821. 16172094 10.1104/pp.105.066761 PMC1183373

[81] Levy A, Guenoune-Gelbart D, Epel BL (2007) Beta-1,3-Glucanases: Plasmodesmal Gate Keepers for Intercellular Communication. Plant Signal Behav 2(5): 404–407. 19704615 10.4161/psb.2.5.4334 PMC2634228

[82] Epel BL (2009) Plant viruses spread by diffusion on ER-associated movement-protein-rafts through plasmodesmata gated by viral induced host beta-1,3-glucanases. Semin Cell Dev Biol 20(9): 1074–1081. 19501662 10.1016/j.semcdb.2009.05.010

[83] Guerra-Peraza O, Kirk D, Seltzer V, Veluthambi K, Schmit AC, et al (2005) Coat proteins of Rice tungro bacilliform virus and Mungbean yellow mosaic virus contain multiple nuclear-localization signals and interact with importin alpha. J Gen Virol 86(Pt 6): 1815–1826. 10.1099/vir.0.80920-0 15914861

[84] Zhong R, Ye ZH (2009) Transcriptional regulation of lignin biosynthesis. Plant Signal Behav 4(11): 1028–1034. 19838072 10.4161/psb.4.11.9875 PMC2819510

[85] Jin H, Li S, Villegas A Jr (2006) Down-regulation of the 26S proteasome subunit RPN9 inhibits viral systemic transport and alters plant vascular development. Plant Physiol 142(2): 651–661. 16905670 10.1104/pp.106.083519 PMC1586039

[86] Camborde L, Planchais S, Tournier V, Jakubiec A, Drugeon G, et al (2010) The ubiquitin-proteasome system regulates the accumulation of Turnip yellow mosaic virus RNA-dependent RNA polymerase during viral infection. Plant Cell 22(9): 3142–3152. 20823192 10.1105/tpc.109.072090 PMC2965540

[87] Wang W, Vinocur B, Shoseyov O, Altman A (2004) Role of plant heat-shock proteins and molecular chaperones in the abiotic stress response. Trends Plant Sci 9(5): 244–252. 15130550 10.1016/j.tplants.2004.03.006

[88] Aranda MA, Escaler M, Wang D, Maule AJ (1996) Induction of HSP70 and polyubiquitin expression associated with plant virus replication. Proc Natl Acad Sci U S A 93(26): 15289–15293. 8986804 10.1073/pnas.93.26.15289 PMC26397

[89] Li G, Zhang J, Tong X, Liu W, Ye X (2011) Heat shock protein 70 inhibits the activity of Influenza A virus ribonucleoprotein and blocks the replication of virus in vitro and in vivo. PLoS One 6(2): e16546. 21390211 10.1371/journal.pone.0016546 PMC3044721

[90] Scarpeci TE, Zanor MI, Valle EM (2008) Investigating the role of plant heat shock proteins during oxidative stress. Plant Signal Behav 3(10): 856–857. 19704521 10.4161/psb.3.10.6021 PMC2634396

[91] Escaler M, Aranda MA, Thomas CL, Maule AJ (2000) Pea embryonic tissues show common responses to the replication of a wide range of viruses. Virology 267(2): 318–325. 10662627 10.1006/viro.1999.0119

[92] Sun W, Bernard C, van de Cotte B, Van Montagu M, Verbruggen N (2001) Aa-HSP17.6A, encoding a small heat-shock protein in Arabidopsis, can enhance osmotolerance upon overexpression. Plant J 27: 407–415. 11576425 10.1046/j.1365-313x.2001.01107.x

[93] Kim DH, Xu ZY, Na YJ, Yoo YJ, Lee J, et al (2011) Small heat shock protein Hsp17.8 functions as an AKR2A cofactor in the targeting of chloroplast outer membrane proteins in Arabidopsis. Plant Physiol 157: 132–146. 21730198 10.1104/pp.111.178681 PMC3165864

[94] Kanzaki H, Saitoh H, Ito A, Fujisawa S, Kamoun S, et al (2003) Cytosolic HSP90 and HSP70 are essential components of INF1-mediated hypersensitive response and non-host resistance to Pseudomonas cichorii in Nicotiana benthamiana. Mol Plant Pathol 4(5): 383–391. 20569398 10.1046/j.1364-3703.2003.00186.x

